# The combination of CUDC-907 and gilteritinib shows promising in vitro and in vivo antileukemic activity against FLT3-ITD AML

**DOI:** 10.1038/s41408-021-00502-7

**Published:** 2021-06-07

**Authors:** Xinan Qiao, Jun Ma, Tristan Knight, Yongwei Su, Holly Edwards, Lisa Polin, Jing Li, Juiwanna Kushner, Sijana H. Dzinic, Kathryn White, Jian Wang, Hai Lin, Yue Wang, Liping Wang, Guan Wang, Jeffrey W. Taub, Yubin Ge

**Affiliations:** 1 National Engineering Laboratory for AIDS Vaccine, School of Life Sciences, Jilin University, Changchun, China; 2 Department of Pediatrics, Wayne State University School of Medicine, Detroit, MI USA; 3 Division of Pediatric Hematology and Oncology, Department of Pediatrics, Children’s Hospital of Michigan, Detroit, MI USA; 4 Department of Oncology, Wayne State University School of Medicine, Detroit, MI USA; 5 Molecular Therapeutics Program, Karmanos Cancer Institute, Wayne State University School of Medicine, Detroit, MI USA; 6 Department of Pathology, Wayne State University School of Medicine, Detroit, MI USA; 7 Department of Hematology and Oncology, The First Hospital of Jilin University, Changchun, P.R. China; 8 Department of Pediatric Hematology and Oncology, The First Hospital of Jilin University, Changchun, P.R. China

**Keywords:** Acute myeloid leukaemia, Drug development

## Abstract

About 25% of patients with acute myeloid leukemia (AML) harbor FMS-like tyrosine kinase 3 (FLT3) internal tandem duplication (ITD) mutations and their prognosis remains poor. Gilteritinib is a FLT3 inhibitor approved by the US FDA for use in adult FLT3-mutated relapsed or refractory AML patients. Monotherapy, while efficacious, shows short-lived responses, highlighting the need for combination therapies. Here we show that gilteritinib and CUDC-907, a dual inhibitor of PI3K and histone deacetylases, synergistically induce apoptosis in FLT3-ITD AML cell lines and primary patient samples and have striking in vivo efficacy. Upregulation of FLT3 and activation of ERK are mechanisms of resistance to gilteritinib, while activation of JAK2/STAT5 is a mechanism of resistance to CUDC-907. Gilteritinib and CUDC-907 reciprocally overcome these mechanisms of resistance. In addition, the combined treatment results in cooperative downregulation of cellular metabolites and persisting antileukemic effects. CUDC-907 plus gilteritinib shows synergistic antileukemic activity against FLT3-ITD AML in vitro and in vivo, demonstrating strong translational therapeutic potential.

## Introduction

Acute myeloid leukemia (AML) is an aggressive, rapidly progressive malignancy. In adults, it constitutes approximately one-third of all leukemia diagnoses and leukemia-related deaths^1^ and comprises ~1 out of every 5 acute leukemias in children, but over half of the deaths among children with acute leukemia^2^. The 5-year survival rates among afflicted children remain unacceptably low, at ~65%, while in adults the 5-year survival rate is even worse at just 27%^3^.

FMS-like tyrosine kinase 3 (FLT3) is a receptor tyrosine kinase expressed on the cell surface of early hematopoietic progenitor cells, where it regulates their normal growth and development^4^. Internal tandem duplication (ITD) mutations in FLT3’s juxtamembrane domain are found in approximately one-quarter of AML cases and result in constitutive FLT3 activation^4^. The presence of FLT3-ITD mutations confers an extremely poor prognosis, due to the activation of multiple downstream survival pathways including MAPK/ERK, PI3K/AKT, and JAK/STAT^4,5^. AXL is also a receptor tyrosine kinase, the activator of which is growth arrest-specific gene 6^6^. Activation of AXL, like FLT3, promotes cell survival/growth via enhancement of the activity of its target pathways^6,7^, and its expression is also inversely correlated with survival and confers a poorer prognosis^8^.

Gilteritinib (ASP-2215) is an orally available, synthetic inhibitor of FLT3 and AXL and was shown to demonstrate potent anti-FLT3 activity in both preclinical^9^ and clinical^10^ setting. Based on interim analysis of the now complete ADMIRAL trial (NCT02421939)^11,12^, gilteritinib was approved by the Food and Drug Administration (FDA) in November 2018 for treatment of adult patients with relapsed/refractory AML and a FLT3 mutation. The emergence of resistance to FLT3 inhibition has been documented to occur in a variety of mechanisms, including overexpression and/or the development of novel/secondary mutations of FLT3^13^. CUDC-907 is an orally available, dual inhibitor of PI3K and histone deacetylases (HDACs). It is currently being investigated in solid tumors, multiple myeloma, and lymphoma in both phase I and phase II trials. CUDC-907 has been granted a Fast Track designation by the FDA for use in adults with relapsed/refractory diffuse large B-cell lymphoma (DLBCL). We previously found that CUDC-907 could downregulate FLT3^14^. This prompted us to hypothesize that downregulation of FLT3 by CUDC-907 would enhance the antileukemic activity of gilteritinib.

In this study, we determined the combined antileukemic activity of CUDC-907 and gilteritinib against FLT3-ITD AML and the underlying molecular mechanism. Combination therapy with gilteritinib and CUDC-907 potently and synergistically induced apoptosis in vitro in FLT3-ITD positive AML cell lines and primary patient samples. This action occurred via a combination of FLT3 downregulation, inhibition of the MAPK/ERK and JAK/STAT pathways, reduction of Mcl-1 and c-Myc, induction of BIM, and downregulation of cellular metabolites. In vivo studies in murine models demonstrated the combination to be well tolerated and resulted in significantly prolonged survival, highlighting the strong translational therapeutic potential of this combination therapy in FLT3-ITD AML.

## Materials and methods

### Drugs

Gilteritinib (ASP-2215), CUDC-907 (Fimepinostat), AZD1480, MG-132, cytarabine (AraC), Lys05, and Z-VAD-FMK were purchased from Selleck Chemicals (Houston, TX, USA).

### Cell cultures

The FLT3-ITD AML cell lines MOLM-13 and MV4-11 were purchased from AddexBio (San Diego, CA, USA; 2012) and American Type Culture Collection (ATCC, Manassas, VA, USA; 2006), respectively. FLT3 wild-type (FLT3-wt) AML cell line THP-1 was purchased from ATCC (2014 and 2002, respectively), while OCI-AML3 was purchased from the German Collection of Microorganisms and Cell Cultures (Braunschweig, Germany; 2011). All cell lines were cultured in RPMI 1640 media (except OCI-AML3, which was cultured in alpha-MEM), with 10–20% fetal bovine serum (CLARK Bioscience, Claymont, DE, USA), plus 2 mM L-glutamine, 100 U/mL penicillin, and 100 μg/mL streptomycin, in a humidified, 5%CO_2_/95% environment at 37 °C. All lines were tested monthly for mycoplasma utilizing the PCR method described by Uphoff and Drexler^15^ and were authenticated in 2017 at the Karmanos Cancer Institute’s Genomics Core via the PowerPlex® 16 System (Promega, Madison, WI, USA).

### Clinical samples

Primary AML patient samples and human umbilical cord blood samples were obtained from the First Hospital of Jilin University. Normal peripheral blood mononuclear cells (PBMCs) were donated by healthy individuals. Informed consent was obtained in all cases as per the Declaration of Helsinki. Both the study and patient sample collection were approved by the Human Ethics Committee of The First Hospital of Jilin University. All AML patient samples were screened for the following gene mutations via PCR amplification and automated DNA sequencing: *FLT3-ITD*, *NPM1*, *C-kit*, *CEBPA*, *IDH1*, *IDH2*, *SF3B1*, *TP53*, *ZRSR2*, *GATA2*, *KMT2A*, *SH2B3*, *TCF*, *TET2*, *RUNX1*, and *DNMT3A*. Cytogenetics and detection of fusion genes via real-time PCR were also performed, as previously described^16,17^. Characteristics of the individual AML patients are listed in Supplementary Table S1. Primary patient samples were purified with Ficoll-Hypaque density centrifugation, and then cultured in RPMI 1640 media plus 20% fetal bovine serum, ITS Solution (Sigma-Aldrich, St Louis, MO, USA), and 20% supernatant of the 5637 bladder cancer cell line (to provide sources of granulocyte–macrophage colony-stimulating factor, granulocyte colony-stimulating factor, interleukin-1 beta, macrophage colony-stimulating factor, and stem cell factor)^16,18,19^.

### Annexin V/propidium iodide (PI) staining and flow cytometry analyses

Cells were treated with the indicated drug(s) for up to 24 h. Flow cytometry analysis was performed utilizing the Annexin V/PI Apoptosis kit (Beckman Coulter, Brea, CA, USA), as previously described^20^. Results are displayed as the percentage of annexin V positive cells, with all cell line experiments repeated in triplicate independently; displayed data are from one representative experiment. Experiments which utilized primary patient samples were performed once in triplicate, due to limitations in sample availability. The extent and direction of the antileukemic interaction between the two agents were determined by calculating the combination index (CI) using CompuSyn software (Combosyn Inc., Paramus, NJ, USA), in which CI < 1, CI = 1, and CI > 1 are respectively indicative of synergistic, additive, and antagonistic effects^20,21^.

### Western blots

Following lysis of the cells in the presence of protease and phosphatase inhibitors (Roche Diagnostics, Indianapolis, IN, USA), whole-cell lysates underwent SDS-polyacrylamide gel electrophoresis, with electrophoretic transfer onto polyvinylidene difluoride membranes (Thermo Fisher Scientific, Rockford, IL, USA), and immunoblotted using anti-Mcl-1 (16225-1-AP), -Bcl-2 (12789-1-AP), -Bcl-xL (66020-1-Ig), -Bax (50599-2-Ig), -β-actin (66009-1-Ig), -ERK (16443-1-AP) (Proteintech, Rosemont, IL, USA), -p-AKT (T308; AF0832), -p-AKT (S473; AF0016) (Affinity Biosciences, Zhenjiang, Jiangsu, China), -Bim (2819), -cf-Caspase 3 (9661), -p-STAT5(Y694; 9359 S) (Cell Signaling Technologies, Danvers, MA, USA), -Bak (ab69404), -p-ERK(T202/Y204; ab4819), -AKT (ab8805), -FLT3 (ab245116) (Abcam, Cambridge, MA, USA), as previously described^22,23^. The Odyssey Infrared Imaging System (Li-Cor, Lincoln, NE, USA) was used to visualize immunoreactive proteins, as described by the manufacturer. Western blots were repeated, at a minimum three times, and one representative blot is displayed. The Odyssey V3.0 program (Li-Cor) was used to perform densitometry measurements, normalized to β-actin, and calculated as fold-change compared to the corresponding vehicle control.

### Ectopic overexpression of Mcl-1 and shRNA knockdown of Bim, Bax, and Bak

The pMD-VSV-G and delta 8.2 plasmids were gifts from Dr. Dong at Tulane University. Red fluorescent protein (RFP) and Mcl-1 cDNA lentiviral constructs were purchased from Dharmacon (Lafayette, CO, USA). Bak, Bax, Bim, and non-target negative control (NTC) shRNA lentiviral constructs were purchased from Sigma-Aldrich. Lentivirus production and transduction were carried out as previously described^24^.

### Quantification of gene transcripts by real-time RT-PCR

Using TRIzol (Thermo Fisher Scientific), total RNA was extracted. cDNAs were prepared using 2 μg of total RNA, random hexamer primers, and a RT-PCR Kit (Thermo Fisher Scientific), and subsequently purified using the QIAquick PCR Purification Kit (Qiagen, Germantown, MD, USA), as described previously^16,21^. Quantification of *FLT3* transcripts was done via the TaqMan probe Hs00174690_m1 (Thermo Fisher Scientific) and a LightCycler 480 real-time PCR machine (Roche Diagnostics), as per manufacturer’s instructions. Results of real-time PCR are presented as the mean of three independent experiments, normalized to *GAPDH* transcripts (TaqMan probe Hs02786624_g1). Fold changes were calculated using the comparative Ct method^25^.

### Leukemia xenograft model

The in vivo efficacy study was conducted following approval by Wayne State University’s Institutional Animal Care and Use Committee. Triple transgenic immunocompromised NSG-SGM3 female mice (NSGS, JAX#0103062; non-obese diabetic scid gamma (NOD.Cg-Prkdc^scid^ Il2rg^tm1Wjl^) Tg(CMV-IL3, CSF2, KITLG)1Eav/MloySzJ) at 8 weeks of age were purchased from Jackson Laboratory (Bar Harbor, ME, USA). On day 0, mice were non-selectively pooled and 1 × 10^6^ MV4-11 cells were injected intravenously (0.2 mL/injection). On day 3, mice were randomly distributed into four groups: vehicle control (*n* = 5), gilteritinib (40 mg/kg; *n* = 5), CUDC-907 (100 mg/kg; *n* = 5), or combination (gilteritinib 40 mg/kg plus CUDC-907 100 mg/kg; *n* = 6). All drugs and vehicle control were administered orally (PO), beginning on day 3, formulated in diluent consisting of 3% ethanol (200 proof), 1% Tween-80 (polyoxyethylene^20^ sorbitan monooleate), and 96% sterile USP cell grade water (v/v). The CUDC-907 was administered daily for 5 days on, followed by 2 days off, for a total of 4 cycles while gilteritinib was administered daily for 28 days. In the combination treatment arm, CUDC-907 was administered 1–2 h prior to gilteritinib. Neither agent was administered on day 19 due to weight loss nadir of 6.7% sustained by the mice on day 18. The treatment resumed on day 20. Mice were weighed daily during the duration of study and assessed a minimum of twice per day at the onset of leukemia symptoms until the completion of the study. Mice were humanely euthanized upon demonstration of weight loss exceeding 20% from pre-treatment period, hind leg paralysis resulting in reduced/inhibited mobility impacting access to food and/or water or lateral recumbency, inability to urinate, optic nerve involvement (eye symptoms), progressive anemia, development of internal or subcutaneous masses >500 mg. All mice were included for efficacy analysis except for one mouse in the CUDC-907 group euthanized on day 10 due to a drug-independent technical issue.

The pharmacodynamics study was approved by the Animal Ethics Committee of the First Hospital of Jilin University. Immunocompromised B-NDG (NOD-Prkdcscid IL2rgtm1/Bcgen) mice (Biocytogen, Beijing, China) were injected with MV4-11 cells (1 × 10^7^ cells/mouse) intravenously. After 21 days, mice were randomized (5 mice/group) and injected with vehicle control, 100 or 150 mg/kg CUDC-907 once. Twenty-four hours later, all mice were sacrificed, and bone marrow cells were collected. Human cells were enriched using the EasySep Mouse/Human Chimera Isolation Kit (Stem Cell Technologies, Vancouver, BC, Canada). For both in vivo efficacy and pharmacodynamic studies, no blinding methods were followed for drug administration.

### Colony-forming assay

Colony-forming assays were carried out as previously described^14,26,27^. Cells were treated with gilteritinib and CUDC-907, alone or in combination, for 24 h, then washed three times with PBS, plated in MethoCult (catalog number 04434; Stem Cell Technologies), and incubated for 10–14 days, according to the manufacturer’s instructions. Colony-forming units (CFUs) were visualized utilizing an inverted microscope. Colonies containing over 50 cells were counted.

### Targeted metabolomics

MV4-11 cells were treated with vehicle control, 12.5 nM gilteritinib, 12.5 nM CUDC-907, or combined gilteritinib and CUDC-907 for 8 h. Cells were collected, quickly washed with PBS, and cell pellets were stored at −80 °C until analysis. Metabolites were extracted using 80% methanol, as previously described^28^. Cellular concentrations of metabolites (normalized to cellular proteins) were quantitatively determined using a LC-MS/MS-based targeted metabolomics platform that is capable of quantitatively profiling ~250 metabolites, as previously described^28^. Data analysis was performed using MetaboAnalyst (www.MetaboAnalyst.ca, version 4.0). Features (metabolites) with >20% missing values (i.e., below the lower limit of quantitation) were removed from the analysis, while the remaining missing values were replaced by 1/5 of the minimum value of a feature. To meet the normality assumption, individual metabolite concentrations were log-transformed and then autoscaled (mean-centered and divided by the standard deviation of each metabolite). The comparisons of individual metabolites among the control and treatment groups were performed by one-way analysis of variance (ANOVA), followed by Fisher’s least-significant differences post hoc analysis. A false discovery rate (FDR) of <0.05 was considered as statistically significant.

### MTT assays

MTT (3-[4,5-dimethyl-thiazol-2-yl]−2,5-diphenyltetrazoliumbromide; Sigma-Aldrich) assays were performed as previously described^20^.

### Statistical analysis

Two-sample *t*-tests were used to compare differences, and statistical analyses were performed utilizing GraphPad Prism 5.0. Error bars are representative of ±standard error of the mean (s.e.m.); significance was set at *p* < 0.05. Overall probability of survival was estimated via the Kaplan–Meier method, and statistical analysis was then performed using log-rank testing. The variance was similar between the groups that were statistically compared. Further, no statistical methods were utilized to predetermine sample size for both in vivo studies. The intravenous leukemia xenograft MV4-11 model historically has a tumor volume doubling time (Td) under 4 days in our hands, resulting in onset of symptoms within 30 +/−3 days for the untreated control group when implanted at an initial titer of 1 × 10^6^ cells on day 0. With these consistent parameters, a sample size of 5–6 mice has been found sufficient to achieve statistical significance. A lower titer (10^4^) was used as an internal control to ensure the experimental engraftment titer is at least 1 log above take-rate (data not shown).

## Results

### CUDC-907 induces time- and concentration/dose-dependent downregulation of FLT3 in FLT3-ITD AML cell lines through proteasome-dependent mechanism

**Fig. 1:**
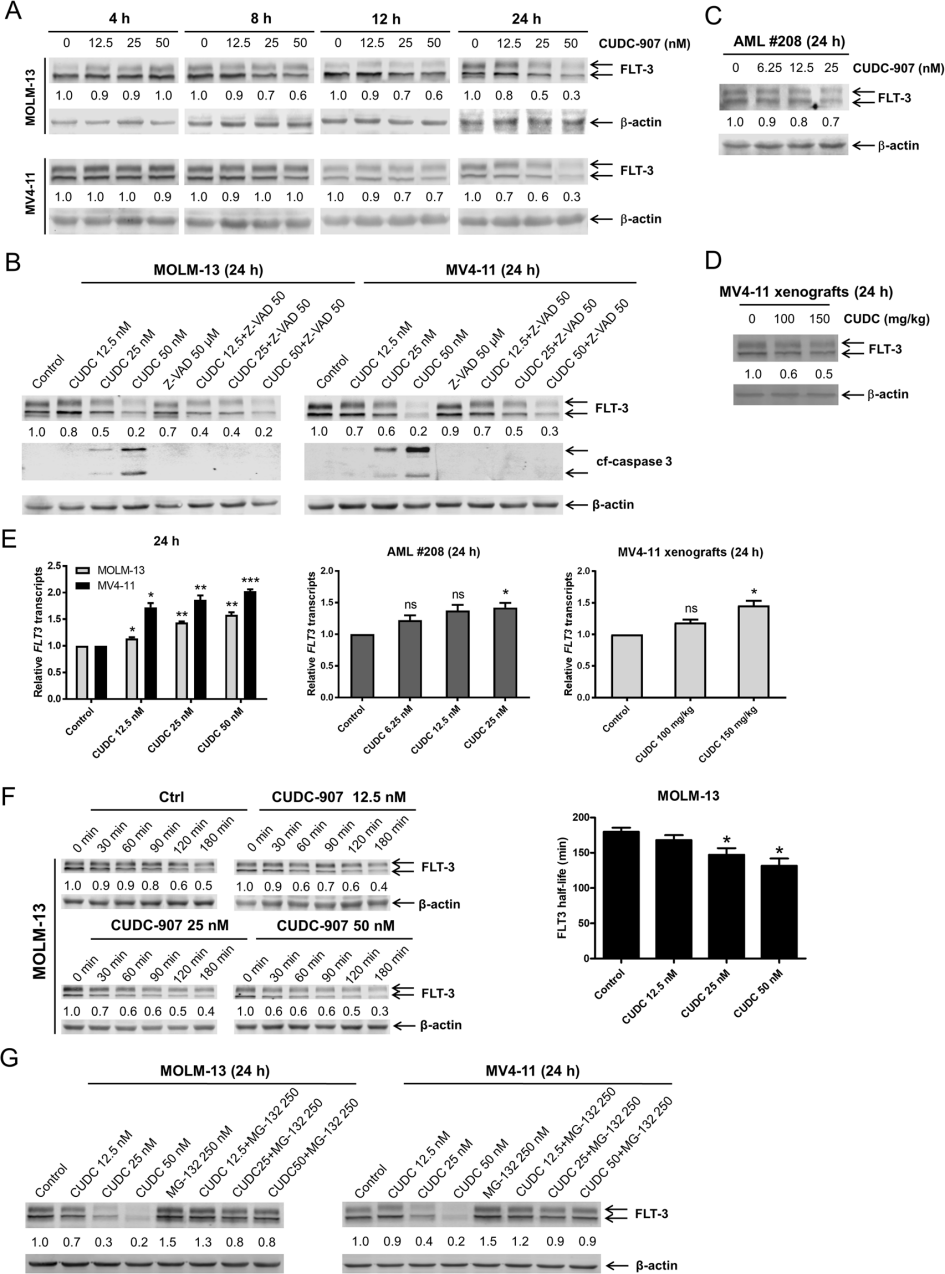
CUDC-907 treatment induces a time- and concentration/dose-dependent downregulation of FLT3 in FLT3-ITD AML cell lines through proteasome-dependent mechanism. **A** FLT3-ITD AML cell lines MOLM-13 and MV4-11 were treated with CUDC-907 for 4, 8, 12, or 24 h, at variable concentrations. Western blots were generated utilizing whole-cell lysates. Representative blots are shown. Densitometry measurements, normalized to β-actin and compared to vehicle control, are displayed below the corresponding blot. **B** FLT3-ITD AML cell lines MOLM-13 and MV4-11 were treated with CUDC-907 and/or Z-VAD-FMK (Z-VAD) for 24 h. Western blot analyses of FLT3 and cleaved-caspase 3 (cf-caspase 3) were performed. Densitometry measurements, normalized to β-actin and compared to vehicle control, are displayed below the corresponding blot. **C** Primary FLT3-ITD positive AML patient sample AML#208 was treated with CUDC-907 for 24 h at variable concentrations. Western blots were generated utilizing whole-cell lysates. Representative blots are shown. Densitometry measurements, normalized to β-actin and compared to vehicle control, are displayed below the corresponding blot. **D** MV4-11 xenograft models were treated once with CUDC-907 at doses of 100 or 150 mg/kg. After 24 h, mouse bone marrow cells were collected and human CD45+ cells were enriched. Whole-cell lysates were subjected to western blotting. Densitometry measurements, normalized to β-actin and compared to vehicle control, are displayed below the corresponding representative blot. **E** FLT3-ITD AML cell lines MOLM-13 and MV4-11, a primary FLT3-ITD positive AML patient sample, and the murine MV4-11 xenograft models were treated with CUDC-907 for 24 h. Total RNA was extracted, and real-time RT-PCR was performed. The relative changes in FLT3 transcripts, normalized to GAPDH, in comparison to control samples were quantified. The displayed results represent the mean of three independent experiments, with fold changes calculated via the comparative Ct method and normalized to GAPDH transcripts. *p < 0.05, **p < 0.01, ***p < 0.001, and ns indicates not significant compared to vehicle control. **F** MOLM-13 cells were treated with CUDC-907 for 12 h, washed, and then treated with cycloheximide for up to 3 h. Western blots were generated utilizing whole-cell lysates. The FLT3 densitometry fold changes were assessed (comparison to vehicle control and normalized to β-actin). Representative blots are shown on the left side, while densitometry measurements are graphed on the right. *p < 0.05 compared to vehicle control. **G** FLT3-ITD AML cell lines MOLM-13 and MV4-11 were treated with CUDC-907 alone or in combination with the proteasome inhibitor MG-132 for 24 h. Western blot analyses of FLT3 were performed, with densitometry results compared to vehicle control and normalized to β-actin.

We first sought to confirm our earlier observation that CUDC-907 downregulates FLT3^14^. Western blot analysis showed a strong concentration-dependent effect of CUDC-907 on FLT3 in vitro in the FLT3-ITD cell lines MV4-11 and MOLM-13 (Fig. 1A). Moreover, time-course experiment results confirmed downregulation of FLT3 as early as 8–12 h, at the higher concentrations, and to persist over a 24 h period (Fig. 1A). Although the pan-caspase inhibitor Z-VAD-FMK completely abolished caspase 3 cleavage induced by CUDC-907, it was unable to block the downregulation of FLT3 caused by the agent (Fig. 1B), demonstrating that FLT3 downregulation was not merely due to caspase activation. Concentration/dose-dependent downregulation of FLT3 also occurred in a primary patient sample ex vivo (Fig. 1C), as well as in the in vivo setting via the MV4-11 xenograft model (Fig. 1D). Real-time RT-PCR quantification of *FLT3* transcripts in MOLM-13 and MV4-11 cell lines, primary patient samples, and MV4-11 murine xenografts post CUDC-907 treatment revealed that CUDC-907 treatment did not reduce *FLT3* transcripts, rather a significant increase or no change was detected (Fig. 1E), showing that the reduction of FLT3 protein is through posttranscriptional mechanism. Next, we assessed FLT3 protein half-life upon exposing MOLM-13 cells to CUDC-907 and identified a significant concentration-dependent reduction, implying reduced protein stability (Fig. 1F). Considering the critical role proteasome and lysosome plays in protein degradation, the impact of proteasome inhibitor MG-132 and the lysosome inhibitor Lys05 on FLT3 downregulation induced by CUDC-907 was determined. The results show that MG-132 could rescue the downregulation of FLT3 (Fig. 1G), while Lys05 could not (Supplementary Figure S1), demonstrating that the downregulation of FLT3 by CUDC-907 is proteasome-dependent.

### Potent antileukemic activity between CUDC-907 and gilteritinib in vitro and in vivo

**Fig. 2:**
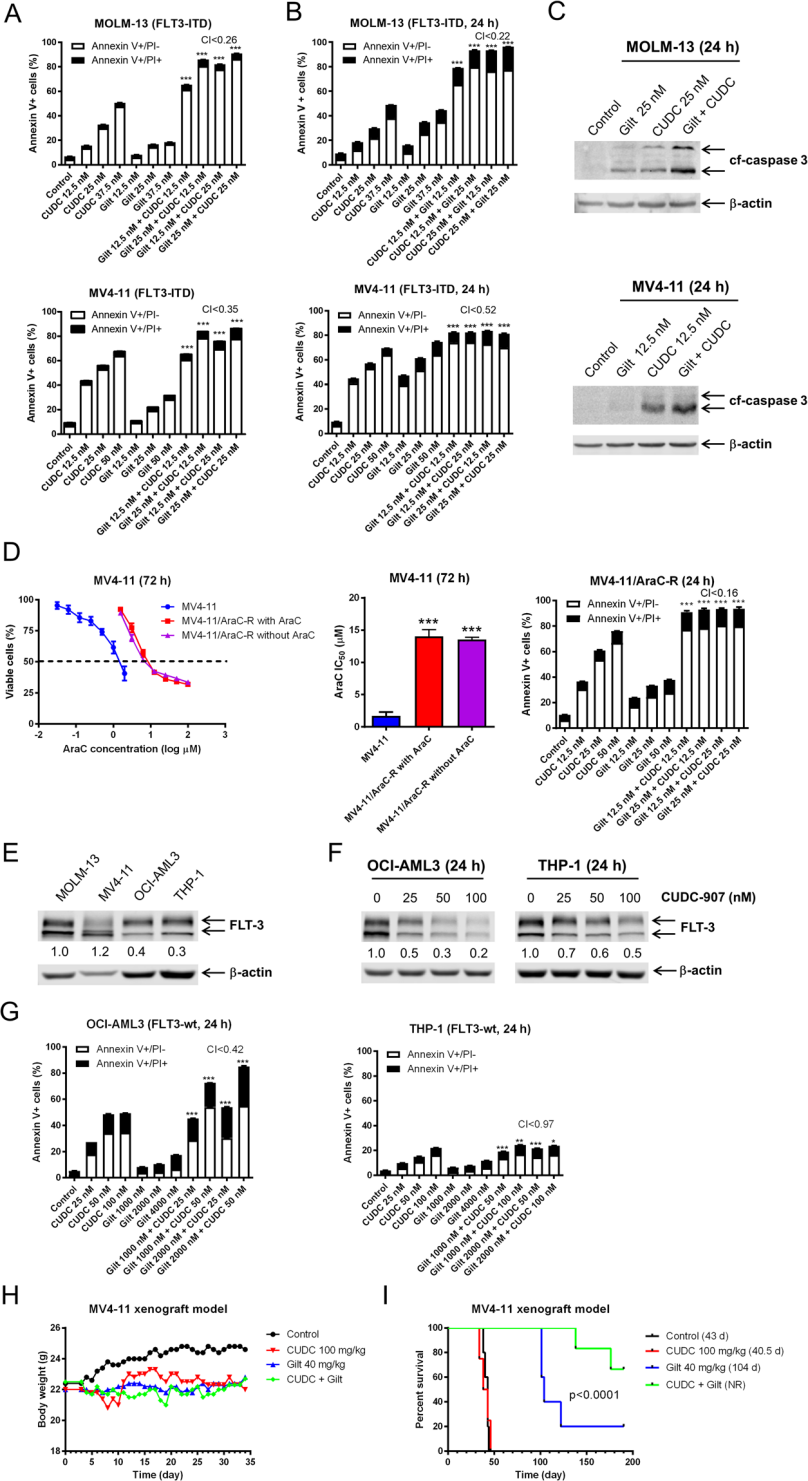
Cooperative antileukemic activity of CUDC-907 plus gilteritinib demonstrated in vitro and in vivo. **A** FLT3-ITD AML cell lines MOLM-13 and MV4-11 were treated with or without CUDC-907, after 12 h gilteritinib was introduced to the indicated samples, for a total CUDC-907 treatment duration of 24 h. Samples were then stained using annexin V/PI, and analysis performed via flow cytometry. Representative experiments are displayed, with combination index (CI) calculated using CompuSyn software. ***p < 0.001 compared to single-drug treatments. Individual CI values are shown in Supplementary Table S4. **B** FLT3-ITD AML cell lines MOLM-13 and MV4-11 were treated with gilteritinib, CUDC-907, both, or neither, for 24 h concurrently, and then annexin V/PI staining and flow cytometry analysis were performed. Representative results from one experiment are displayed. **C** MOLM-13 and MV4-11 cells were treated with gilteritinib, CUDC-907, both, or neither, for 24 h concurrently, and then whole-cell lysates underwent western blot analysis of cleaved-caspase 3 (cf-caspase 3) and β-actin. **D** MV4-11 cells were treated with stepwise increasing concentrations of cytarabine to generate MV4-11 cells with acquired cytarabine resistance (designated MV4-11/AraC-R). MV4-11/AraC-R cells were cultured in the presence or absence of the AraC concentration used to maintain resistance (1100 nM) for 5 days. Then the AraC resistant cells and parental cells were treated with variable concentrations of AraC for 72 h. Viable cells were determined using the MTT assay. Representative curves are shown on the left. IC50s were calculated and are presented on the right. ***p < 0.001 compared to the parental cells. MV4-11/AraC-R cells were treated with vehicle control, CUDC-907, gilteritinib, or in combination for 24 h. Cells were stained with annexin V/PI and subjected to flow cytometry analyses. CI values were calculated using CompuSyn software. Individual CI values are shown in Supplementary Table S4. ***p < 0.001 compared to control and single-drug treatment. **E** Western blots of FLT3 were generated utilizing whole-cell lysates from AML cell lines MOLM-13, MV4-11, OCI-AML3, and THP-1. Densitometry results compared to MOLM-13 and normalized to β-actin are shown. **F** OCI-AML3 and THP-1 cells were treated with CUDC-907 for 24 h. Whole-cell lysates were subjected to western blotting. Densitometry results for FLT3 compared to vehicle control and normalized to β-actin are shown. **G** The FLT3-wt AML cell lines THP-1 and OCI-AML3 were treated with CUDC-907, gilteritinib, both, or neither, for 24 h concurrently, and then underwent annexin V/PI staining and flow cytometry analysis. CI values were calculated using CompuSyn software. Individual CI values are shown in Supplementary Table S4. *p < 0.05, **p < 0.01, and ***p < 0.001 compared to single-drug treatment. **H**, **I** NSGS mice were injected with 1 × 10^6^ MV4-11 cells/mouse via the tail vein on day 0. Mice were then randomized to one of four treatment groups: vehicle control (3% 200 proof ethanol, 1% polyoxyethylene sorbitan monooleate, and USP water; *n* = 5), CUDC-907 (100 mg/kg; *n* = 5), gilteritinib (40 mg/kg; *n* = 5), or combination (100 mg/kg CUDC-907 plus 40 mg/kg gilteritinib; *n* = 6). Treatment was initiated on day 3, with daily gilteritinib dosing and 5 days of daily CUDC-907 dosing followed by 2 days off, for a total of 28 treatment days. Daily body weights were measured and graphed as the mean for each group (**H**). Overall proportion of mice in each treatment group surviving, estimated via Kaplan–Meier method, is shown in panel (**I**). One mouse in the CUDC-907 group was excluded due to a drug-independent technical issue. NR indicates not reached.

The ability of CUDC-907 to downregulate FLT3 indicates that it may enhance the antileukemic activity of gilteritinib. To test this possibility, we treated the FLT3-ITD cell lines MOLM-13 and MV4-11 with clinically achievable concentrations of gilteritinib, CUDC-907, both, or vehicle control. Based on our identification of a time-dependent downregulation of FLT3 as described above, we treated the cells initially with CUDC-907, and then 12 h later with gilteritinib for a total duration of 24 h. We detected a strong synergistic induction of annexin V positive cells (indicative of apoptosis) in both cell lines (MOLM-13 CI < 0.26, MV4-11 CI < 0.35; Fig. 2A). Importantly, annexin V positivity induction remained potent and synergistic when the cell lines were treated simultaneously with the two agents for 24 h (MOLM-13 CI < 0.58, MV4-11 CI < 0.52; Fig. 2B). Further, increased annexin V staining was accompanied by elevated levels of cleaved-caspase 3, confirming cell apoptosis (Fig. 2C).

Since gilteritinib is approved for use in refractory/relapsed AML and new therapies are first tested in this patient population, we next sought to determine if the combination would also synergistically induce apoptosis in AML cells resistant to frontline chemotherapeutic AraC (cytarabine). As shown in Fig. 2D, MV4-11 AraC resistant cells, (MV4-11/AraC-R), had a significantly increased AraC IC_50_ compared to parental MV4-11 cells. To ensure that downstream experimental results were without the interference of AraC, AraC was removed for 5 days prior to subjecting the MV4-11/AraC-R cells to MTT assays. Notably, removal of AraC for 5 days prior had no significant effect on AraC IC_50_ (Fig. 2D). As in the parental cells, the combination treatment synergistically induced apoptosis in MV4-11/AraC-R cells, suggesting that acquired AraC resistance does not confer cross-resistance to combined gilteritinib and CUDC-907 treatment.

To explore the treatment effects of this combination on FLT3 wild-type (FLT3-wt) AML, we first determined the protein level of FLT3 in several AML cell lines. FLT3-wt THP-1 and OCI-AML3 cell lines had substantial FLT3 protein detected, though it was relatively lower than in the FLT3-ITD positive MOLM-13 and MV4-11 cells (Fig. 2E). Treatment of OCI-AML3 and THP-1 cell lines with CUDC-907 resulted in a concentration-dependent decrease of FLT3 protein, but the extent was much greater in OCI-AML3 cells (Fig. 2F). Although the combination treatment also induced apoptosis in THP-1 cells in an additive to synergistic manner, the magnitude was markedly lower. In contrast, strong synergy between the two agents was detected in OCI-AML3 cells (Fig. 2G), demonstrating that the two agents also synergize in inducing apoptosis in certain FLT3-wt AML cells.

To assess the in vivo efficacy of the combination of CUDC-907 and gilteritinib, we developed a mouse xenograft model utilizing the MV4-11 cell line engrafted into NSGS female mice. Mice were randomized into four groups (vehicle control, gilteritinib [40 mg/kg], CUDC-907 [100 mg/kg], or combination). Beginning on day 3 post cell injection, mice received CUDC-907 daily for 5 days on, 2 days off, for a total of 4 cycles, gilteritinib administered daily, both, or vehicle control. The treatment schedule for CUDC-907 was used to mimic the clinical schedule of drug administration. Weight was assessed daily for each mouse (Fig. 2H). One mouse in the CUDC-907 group was excluded due to a drug-independent technical issue. In the CUDC-907 group, maximal weight loss was 5.5%. Gilteritinib was well tolerated, at no point did the mean weight of the group fall more than 0.9% below pre-treatment weight. Combined treatment did result in more sustained weight loss and remained consistently below pre-treatment weight, with a nadir of 6.7% lower on day 18; upon being given a ‘day off’ on day 19, weight loss returned to a more moderate range. Following completion of treatment on day 31, pre-treatment weight was recovered and surpassed on day 34.

Median survival of mice in the vehicle control group was 43 days (38–44 days), while for the CUDC-907 arm median survival was 40.5 days (34–46 days; Fig. 2H). Median survival for the gilteritinib arm was 104 days. In the combination therapy arm, 1 mouse died on day 138 and another died on day 176, and the remaining 4 mice were alive and asymptomatic on day 190 post cell injection (Fig. 2I; median survival was not reached). To rule out the possibility of immune recovery, we implanted 10^6^ leukemia cells IV into these mice; all succumbed to systemic disease as expected, ruling out immune recovery.

### CUDC-907 and gilteritinib synergistically induce apoptosis in primary AML patient samples ex vivo

**Fig. 3:**
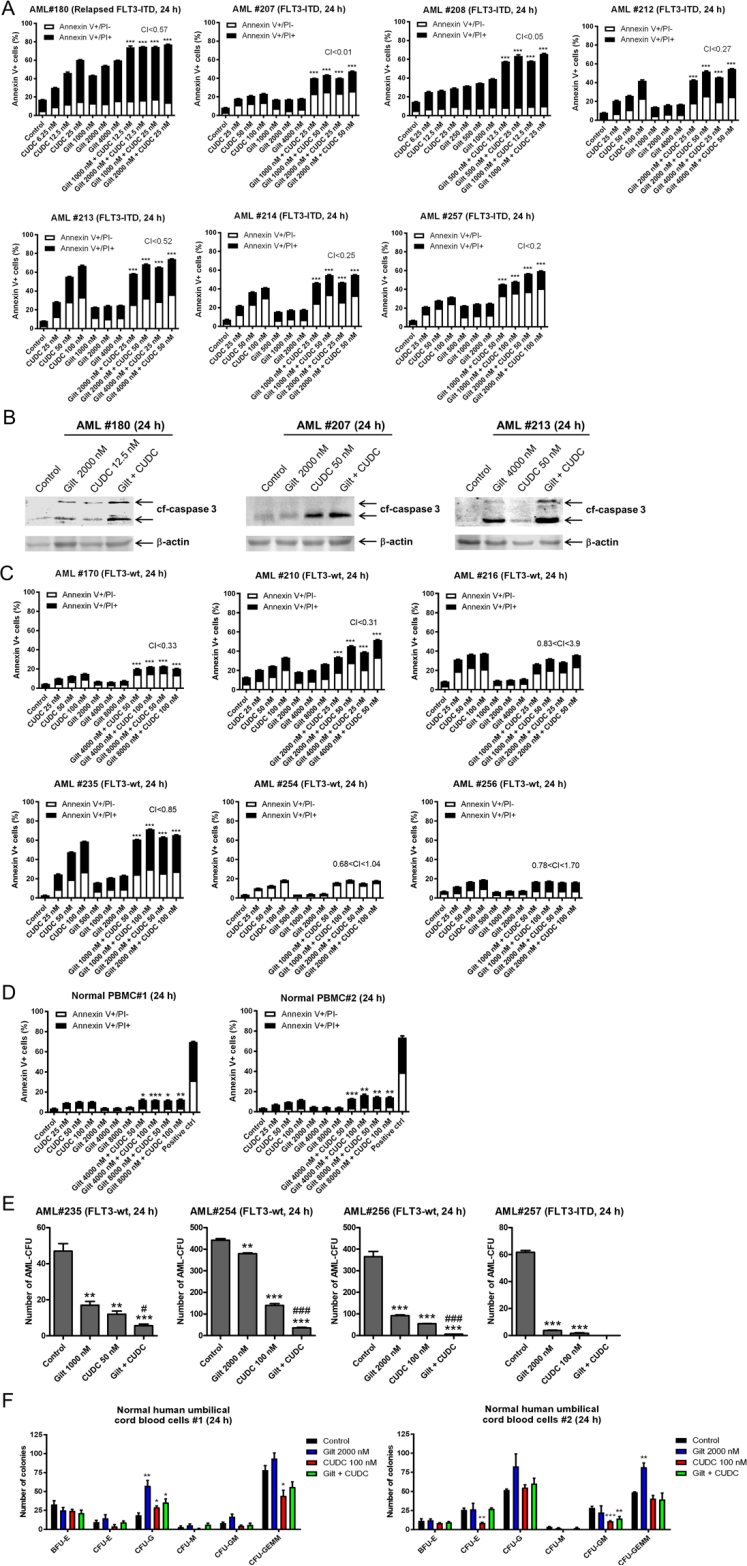
Synergistic antileukemic activity of CUDC-907 plus gilteritinib in primary AML patient samples ex vivo. **A** Primary AML patient samples (FLT3-ITD positive) were treated with gilteritinib, CUDC-907, both, or neither, for 24 h concurrently, and then annexin V/PI staining and flow cytometry analysis was performed. CI values were calculated using CompuSyn software. Individual CI values are shown in Supplementary Table S5. **B** Three primary patient samples (FLT3-ITD positive) were treated with gilteritinib and/or CUDC-907 for 24 h and then whole-cell lysates were subjected to western blot analysis of cleaved-caspase 3 (cf-caspase 3) and β-actin. **C**, **D** Six FLT3-wt AML patient samples (panel **C**) and two samples of normal peripheral blood mononuclear cells (panel **D**) were treated with gilteritinib, CUDC-907, both, or neither, for 24 h concurrently, and then annexin V/PI staining and flow cytometry analysis were performed. CI values were calculated using CompuSyn software. Individual CI values are shown in Supplementary Table S6. *p < 0.05, **p < 0.01, and ***p < 0.001 compared to single-drug treatments. **E**, **F** Primary AML patient samples (panel **E**) and normal human umbilical cord blood cells (panel **F**) were treated with gilteritinib, CUDC-907, both, or vehicle control for 24 h concurrently, and then plated in methylcellulose. The number of leukemic colonies (AML-CFUs), erythroid, and myeloid colonies were counted 10–14 days later. Data are presented as mean ± SEM. *p < 0.05, **p < 0.0,1 and ***p < 0.001 compared to control. ^#^*p* < 0.05 and ^###^*p* < 001 compared to single-drug treatments. Technical triplicates were performed.

To provide further support for the clinical translation of the combination of gilteritinib and CUDC-907, FLT3-ITD positive primary AML patient samples were treated ex vivo. Consistent with the FLT3-ITD AML cell lines, synergistic induction of apoptosis was detected in all 7 primary AML patient samples (CI < 0.6; Fig. 3A). This was accompanied by increased cleavage of caspase 3 (Fig. 3B). In FLT3-wt primary AML patient samples, combined treatment synergistically induced apoptosis in 3 of the 6 samples (though the magnitude of increase over single-drug treatment appears to be minimal), while antagonistic effects were detected in the other 3 samples (Fig. 3C). To assess the effect on non-malignant cells, we treated normal, healthy PBMCs with vehicle control, gilteritinib, CUDC-907, or both for 24 h. In both cases, the percentage of cell death was well below 20% (Fig. 3D).

To determine the effect of the combination on leukemic progenitor cells, colony-forming assays were performed using primary AML patient samples (*n* = 4). Interestingly, in the three FLT3-wt samples, the combination treatment significantly enhanced reduction of colony-forming capacity compared to both monotherapies. In the FLT3-ITD sample, both monotherapy treatments had a significant impact on colony formation, while the combination treatment resulted in no detectable colonies (Fig. 3E). In contrast, gilteritinib did not significantly reduce colony formation of human umbilical cord blood samples (*n* = 2). While CUDC-907 treatment significantly reduced formation of granulocyte and multilineage colonies in sample 1 and erythroid and granulocyte–macrophage colonies in sample 2, there was no further reduction on any of the colony types following combined treatment (Fig. 3F). Taken together, these results suggest a potential therapeutic window for this combination therapy.

### CUDC-907 abrogates gilteritinib-induced FLT3 upregulation and enhances apoptosis induced by gilteritinib

**Fig. 4:**
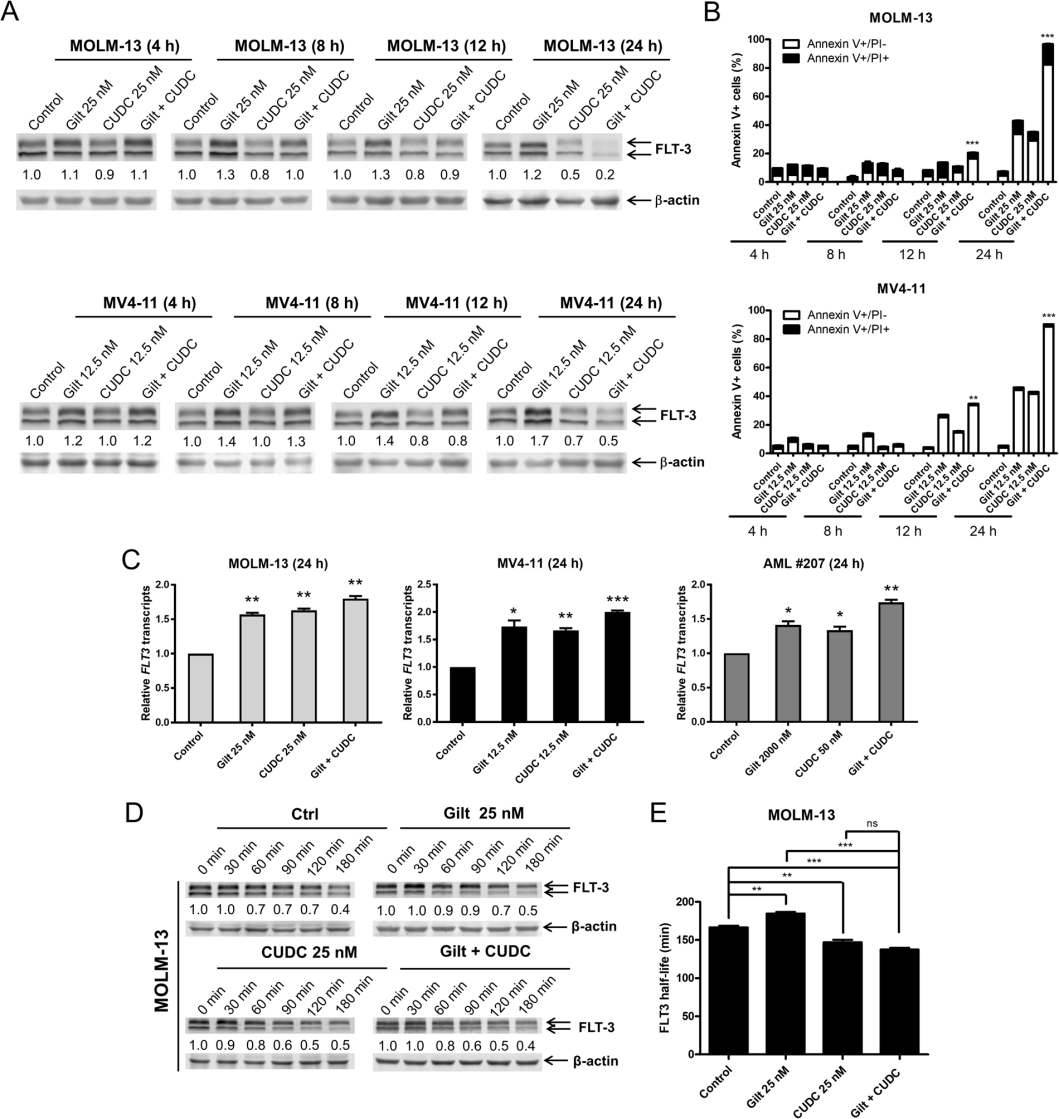
Gilteritinib induces FLT3 upregulation, which is abrogated by CUDC-907. **A** FLT3-ITD AML cell lines MOLM-13 and MV4-11 were treated with gilteritinib, CUDC-907, both, or neither for 4, 8, 12, or 24 h, at concentrations of 12.5 or 25 nM, as indicated. Western blots were generated using whole-cell lysates. One representative blot is shown. Densitometry measurements, compared to vehicle control and normalized to β-actin, are shown below the corresponding blots. **B** MOLM-13 and MV4-11 cells were treated as described in panel A and then stained with annexin V/PI and subjected to flow cytometry analysis. **p < 0.01 and ***p < 0.001 compared to single-drug treatments at the same timepoint. **C** MOLM-13, MV4-11, and primary FLT3-ITD positive AML patient sample AML#207 were treated with gilteritinib, CUDC-907, both, or vehicle control for 24 h at the indicated concentrations. Total RNA was extracted, and *FLT3* transcripts relative to *GAPDH* were determined by real-time RT-PCR. *p < 0.05, **p < 0.01, and ***p < 0.001 compared to vehicle control. **D**, **E** MOLM-13 cells were treated with CUDC-907 and/or gilteritinib for 12 h, washed, and then treated with cycloheximide for up to 3 h. Western blots were generated utilizing whole-cell lysates. The fold changes for the FLT3 densitometry were assessed via comparison to vehicle control and normalized to β-actin. Representative blots are shown in panel (**D**), while densitometry measurements are graphed in panel (**E**). **p < 0.01, ***p < 0.001, and ns indicates not significant.

As has been described previously^9^, gilteritinib is known to induce FLT3, including in vivo in human patients. Accordingly, western blot analysis confirmed that gilteritinib-induced FLT3 within 4–8 h of exposure (Fig. 4A). By 12 h, CUDC-907’s effect, e.g., downregulation of FLT3, began to diminish FLT3 levels in the combination group compared to gilteritinib alone, which was accompanied by significant induction of apoptosis by the combined treatment (Fig. 4B). Interestingly, we found that combined CUDC-907 and gilteritinib treatment increased *FLT3* transcripts compared to the control in the FLT3-ITD cell lines and the primary patient sample. Monotherapy-treated samples also demonstrated similar transcriptional patterns, with relative transcripts higher than the control sample. These results indicate that transcriptional downregulation was not the predominant mechanism by which FLT3 protein was being diminished. In addition, these results suggest that transcriptional regulation may play a role in the upregulation of FLT3 by gilteritinib.

To explore whether the alteration of FLT3 protein levels is related to protein stability, we assessed FLT3 half-life in MOLM-13 cells. The results showed that the gilteritinib treatment increased FLT3 protein stability, meanwhile CUDC-907 could abolish this effect (Fig. 4D, E). These results suggest that gilteritinib may upregulate FLT3 by inducing *FLT3* gene transcription and increasing FLT3 protein stability, while CUDC-907 reduces the stability of FLT3 protein, abolishing the upregulation of FLT3 by gilteritinib, resulting in synergistic antileukemic activity.

### CUDC-907 and gilteritinib cooperatively inactivate ERK and STAT5

**Fig. 5:**
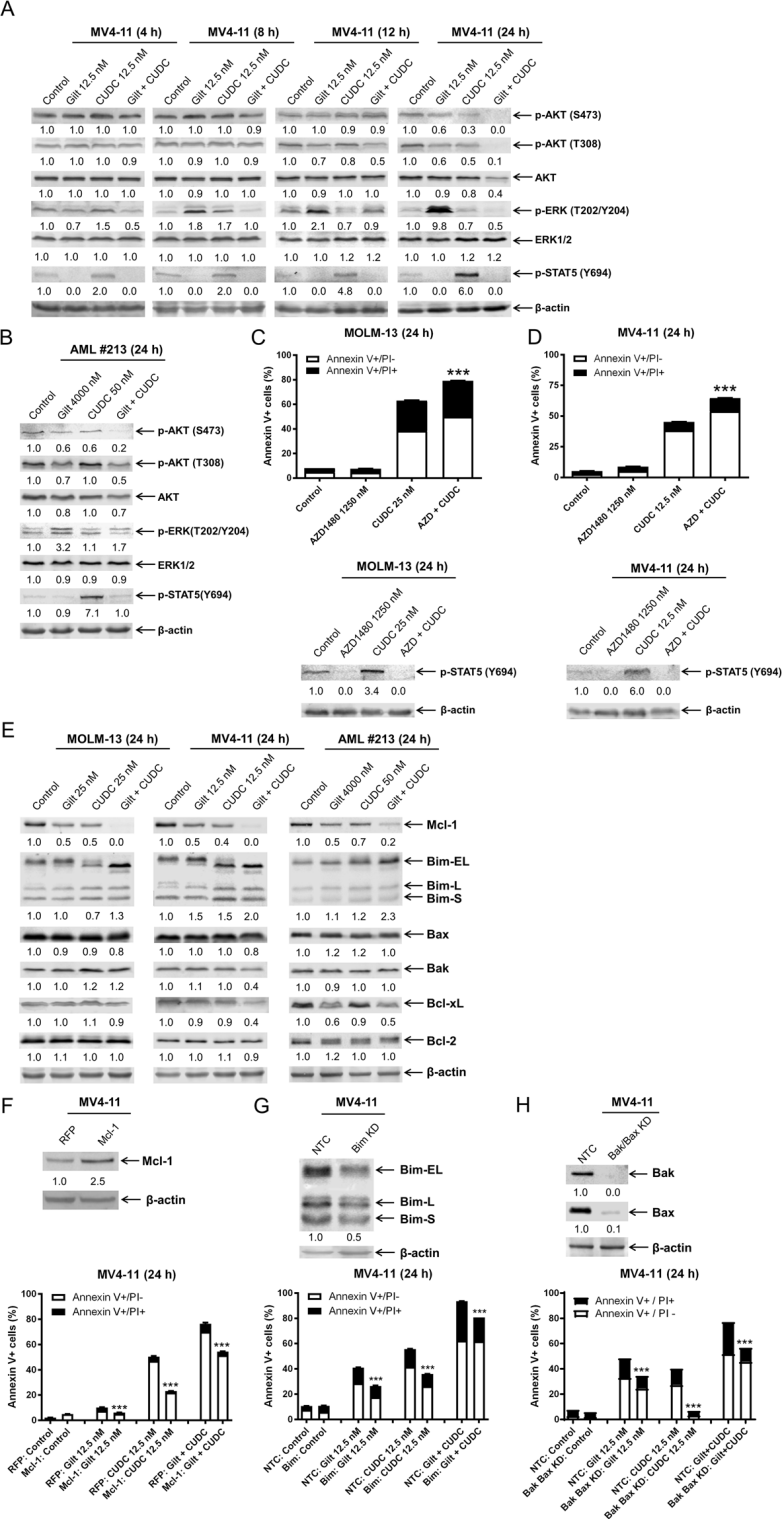
CUDC-907 and gilteritinib induce complimentary and cooperatively altered activity within the MAPK/ERK and JAK2/STAT5 pathways. **A** MV4-11 cells were treated with gilteritinib, CUDC-907, both, or neither for 4, 8, 12, or 24 h. Western blots were generated utilizing whole-cell lysates, with representative blots shown, and densitometry displayed below each blot. Densitometry was assessed via comparison to vehicle control and normalized to β-actin. **B** A primary FLT3-ITD positive AML patient sample was treated with gilteritinib and/or CUDC-907 for 24 h. Densitometry was assessed via comparison to vehicle control and normalized to β-actin. **C**, **D** MOLM-13 and MV4-11 cells were treated using CUDC-907 either with or without AZD1480, a selective JAK2 inhibitor, for 24 h. Flow cytometry analysis of annexin V/PI stained cells is shown in the upper panels and western blot analyses of phosphorylated STAT5 are shown in the lower panels. ***p < 0.001 compared to single-drug treatments. **E** MOLM-13, MV4-11, and primary patient sample AML#213 were treated with gilteritinib, CUDC-907, both, or vehicle control for 24 h. Western blotting was performed to analyze expression of members of the Bcl-2 family. Densitometry was measured via comparison to vehicle control and normalized to β-actin. **F** Mcl-1 overexpression and red fluorescent protein (RFP) control MV4-11 cells were generated using lentivirus particles as described in the “Methods” section. Whole-cell lysates were subjected to western blotting to confirm overexpression (upper panel). The cells were then treated with vehicle control, gilteritinib, CUDC-907, or in combination for 24 h, and then annexin V/PI staining and flow cytometry analysis were performed (lower panel). ***p < 0.001 compared to RFP under the same drug treatment. **G**, **H** shRNA knockdown of Bim, Bak, and Bax, or non-template control (NTC) was performed in MV4-11 cells. Whole-cell lysates were subjected to western blotting (upper panels). Cells were treated with vehicle control, gilteritinib, CUDC-907, or in combination for 24 h. Annexin V/PI staining and flow cytometry analysis results are shown (lower panels). ***p < 0.001 compared to NTC for the same drug treatment.

We next sought to determine the effect of CUDC-907 and gilteritinib on the signaling pathways downstream of FLT3. Both agents alone and in combination reduced phosphorylated AKT (p-AKT) by 24 h (Fig. 5A). Consistent with our previous findings^29^, gilteritinib treatment initially decreased phosphorylated ERK (p-ERK), but levels were substantially increased by 8 h and continued to increase through 24 h. CUDC-907 treatment increased phosphorylated STAT5 (p-STAT5), as early as 4 h after treatment initiation. The increase of p-ERK and p-STAT5 was completely abolished by the drug combination (Fig. 5A). Similar results were obtained in MOLM-13 cells (Supplementary Figure S2) and a primary patient sample (Fig. 5B). These results suggest that the cooperative inactivation of ERK and STAT5 contributes to the synergistic induction of apoptosis by the two agents; while suppression of AKT may also contribute to apoptosis induced by the two agent, the concomitant downregulation of p-AKT and total AKT and significant increase of Annexin V positivity occurring at the later timepoints suggest that it is unlikely to be responsible for the synergy.

Given the reported role of p-ERK induction in gilteritinib resistance in FLT3-ITD AML^29^, the abolishment of the gilteritinib-induced increase of p-ERK by CUDC-907 represents a mechanism responsible for the synergistic antileukemic interaction between the two agents. To determine the role of p-STAT5 in the synergistic antileukemic interactions between the two agents, we combined CUDC-907 with AZD1480 (AZD), a JAK2 inhibitor^30^. Annexin V/PI staining and flow cytometry analyses confirmed cooperative induction of apoptosis by AZD1480 combined with CUDC-907 in both MOLM-13 and MV4-11 cell lines (Fig. 5C, D). Western blot analysis confirmed that AZD inhibited JAK2 and its combination with CUDC-907 completely abolished the induction of p-STAT5 by CUDC-907 (Fig. 5C, D). These results show that activation of STAT5 represents a mechanism of CUDC-907 resistance and can be completely abolished by gilteritinib, resulting in synergistic induction of apoptosis.

### CUDC-907 and gilteritinib cooperate in downregulation of Mcl-1 and upregulation of Bim, leading to apoptosis

Based on the roles the ERK pathway plays in the regulation of Bcl-2 family proteins and the role the STAT5 pathway plays in regulating Mcl-1 in the context of FLT3-ITD^31–34^, we assessed alterations in the Bcl-2 family of proteins in the FLT3-ITD AML cell lines and a primary patient sample, upon exposure to CUDC-907, gilteritinib, both, or vehicle control. While both agents downregulated anti-apoptotic protein Mcl-1, combination treatment resulted in further decrease. A cooperative increase in the amount of pro-apoptotic protein Bim was also observed to occur upon combined treatment (Fig. 5E). As Bim and Mcl-1 were the primary Bcl-2 family members in which we observed alterations in levels, we performed a time-course experiment and found that Mcl-1 downregulation began as early as 4 h after exposure to gilteritinib, though enhanced downregulation was observed only at 24 h (Supplementary Figure S3). Bim levels increased after 4–12 h of combined treatment.

To determine the role of Mcl-1 and Bim in apoptosis induced by the combination of gilteritinib and CUDC-907, we overexpressed Mcl-1 and knocked down Bim, respectively, in MV4-11 cells. Mcl-1 overexpression partially rescued cells from gilteritinib and CUDC-907 treatment, both alone and in combination (Fig. 5F). Similarly, Bim knockdown partially rescued MV4-11 cells from gilteritinib and CUDC-907, both alone and in combination (Fig. 5G). These results suggest that Mcl-1 and Bim play an important role in apoptosis induced by CUDC-907 and gilteritinib. Double knockdown of Bax and Bak resulted in near-complete rescue of cells treated with CUDC-907, while gilteritinib treatment and the combination treatment resulted in partial rescue (Fig. 5H), demonstrating that combination treatment induces apoptosis at least partially through the intrinsic apoptosis pathway.

### CUDC-907 and gilteritinib rapidly reduce c-Myc and cellular metabolites

**Fig. 6:**
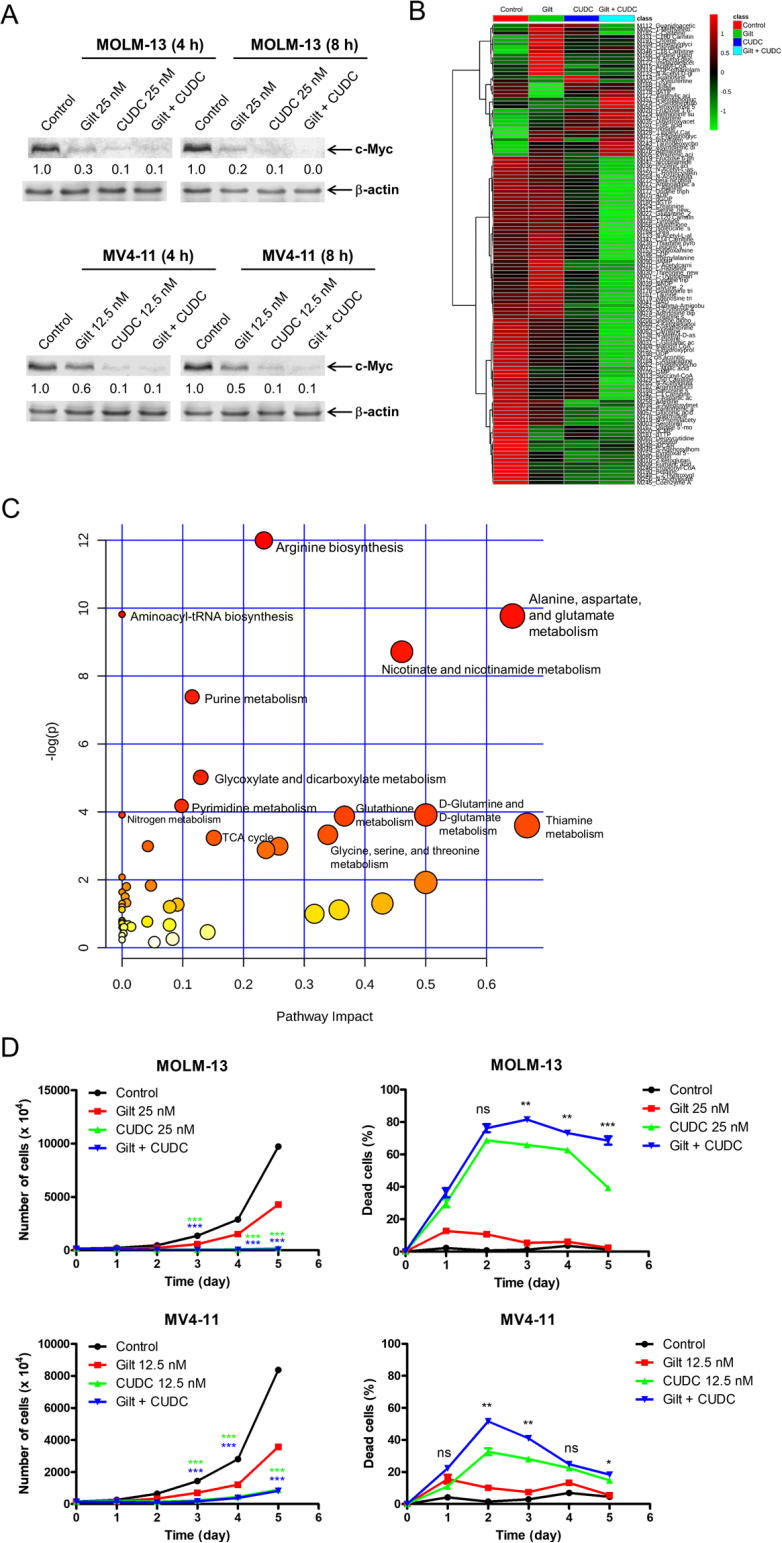
CUDC-907 and gilteritinib treatment depletes intracellular metabolites. **A** MOLM-13 and MV4-11 were treated with gilteritinib, CUDC-907, both, or vehicle control for 4 or 8 h. Western blots were generated utilizing whole-cell lysates, with representative blots shown, and densitometry displayed below each blot. Densitometry was assessed via comparison to vehicle control and normalized to β-actin. **B** MV4-11 cells were treated with gilteritinib and/or CUDC-907 for 8 h, at concentrations of 12.5 nM. Cells were collected, washed with PBS, and cell pellets were stored at −80 °C. Metabolites were quantitatively profiled using LC-MS/MS-based targeted metabolomics platform in the Karmanos Cancer Institute Pharmacology Core. Data were analyzed using www.MetaboAnalyst.ca, version 4.0. Heatmap analysis of the 125 metabolites with significantly different expression levels is shown. **C** Pathway analysis of the 125 metabolites with significantly different expression levels is shown. **D** MOLM-13 and MV4-11 cells were treated with vehicle control, gilteritinib, CUDC-907, or in combination for 8 h, and then the cells were washed and resuspended in fresh media without gilteritinib or CUDC-907. The cells were counted daily with trypan blue staining. Total number of cells are graphed in the left panels, while the percentage of dead cells (trypan blue positive cells) is graphed in the right panels. *p < 0.05, **p < 0.01, ***p < 0.001, and ns indicates not significant (left panels: compared to vehicle control and gilteritinib treatment; right panels: compared to vehicle control and single-drug treatments).

The lack of complete rescue of MV4-11 cells from the combined treatment of CUDC-907 and gilteritinib by Bak/Bax double knockdown indicates that other mechanisms exist besides induction of intrinsic apoptosis. We previously showed that CUDC-907 downregulates c-Myc^14^, which is a key regulator of cellular metabolism^35–39^. Thus, we hypothesized that CUDC-907 and gilteritinib cooperate in downregulating cellular metabolic activity, contributing to the synergistic antileukemic activity. To test this possibility, we first examined the protein levels of c-Myc following treatment with gilteritinib and CUDC-907. Both monotherapies substantially decreased the levels of c-Myc as early as 4 h, while the combination treatment reduced c-Myc to levels similar to CUDC-907 treatment alone (Fig. 6A). We then performed targeted metabolomics analysis to monitor the fluctuation of cellular metabolites in MV4-11 cells following treatment with gilteritinib and CUDC-907, alone and in combination, for 8 h, a treatment length that did not induce apoptosis in the cells (Fig. 4B). There were 125 significantly changed metabolites among the different treatment groups (Fig. 6B and Supplementary Table S2). Pathway analysis revealed that the significantly changed metabolites were mainly involved in amino acid metabolism, fatty acid metabolism, and the TCA cycle (Fig. 6C, Supplementary Table S3, and Supplementary Figures S4–S6). Notably, the combination treatment also resulted in significant decrease of nucleotides including ATP (Supplementary Figure S7).

To determine if CUDC-907 and gilteritinib treatment has persisting effects on cell proliferation and survival, drug washout experiments were performed. MOLM-13 and MV4-11 cells were treated with vehicle control, gilteritinib, CUDC-907, or in combination for 8 h, and then the cells were washed and resuspended in fresh media without gilteritinib or CUDC-907. The cells were counted daily using trypan blue. Five days after drug washout, both CUDC-907 and the combination treatment resulted in significant decrease of the total number of cells compared to gilteritinib or vehicle control (Fig. 6D, left panels). Although there was no significant difference in total number of cells between the CUDC-907 and combination treatment, the percent of dead cells in the combination treatment was significantly higher compared to individual drug treatment and the vehicle treatment 2 to 3 days after drug washout (Fig. 6D, right panels). Taken together, these results show that the combined drug effects persist after washout.

## Discussion

In our previous study, we reported that FLT3-ITD positive AML patient samples were more sensitive to CUDC-907 than FLT3-wt AML patient samples ex vivo^14^. In addition, we found that CUDC-907 downregulated FLT3. Building upon this, here we confirmed downregulation of FLT3 in a primary AML patient sample and in the in vivo setting via the MV4-11 xenograft NSG mouse model (Fig. 1C, D). HDACIs have been reported to cause proteasomal degradation of FLT3 via upregulation of ubiquitin conjugase^40^ and inhibition of HSP90^41,42^. Consistent with this, we found that CUDC-907 downregulates FLT3 via proteasomal degradation (Fig. 1). In agreement with previous reports^13^, we found that gilteritinib treatment upregulates *FLT3* transcripts and protein stability. While transcripts remain elevated following combined treatment, FLT3 protein stability is reduced (Fig. 4D, E).

We and others have reported that FLT3 inhibition can cause ERK activation^29,43–45^. In addition, we have reported that CUDC-907 inactivates ERK^14^. In this study, we identified that CUDC-907 treatment activates STAT5 while gilteritinib inactivates it (Fig. 5 and Supplementary Figure S2). In combination, gilteritinib and CUDC-907 reciprocally overcome these mechanisms of resistance. In agreement with prior literature^46^, we found that the anti-apoptotic protein Mcl-1 is downregulated via exposure to gilteritinib. Moreover, gilteritinib also appears to induce the pro-apoptotic protein Bim. Our results concerning CUDC-907, e.g., Bim-induction and Mcl-1 downregulation, are consistent with prior known alterations resulting from combined PI3K/HDAC inhibition^47–49^. These alterations were accentuated by combination therapy with both agents and at least partially contributed to the intrinsic apoptosis induced by the combination. However, double knockdown of Bak and Bax only rescued a fraction of cells from the combination treatment, indicating that other mechanisms exist.

Another important factor contributing to the synergy between CUDC-907 and gilteritinib is metabolism. c-Myc is downregulated by CUDC-907, as previously reported^14^. c-Myc positively regulates glycolysis and fatty acid oxidation^35,36^. Most glycolytic enzyme–encoding genes are regulated at some level by c-Myc (reviewed in ref. ^37^). c-Myc targets over 400 mitochondrial genes and loss of Myc has been shown to reduce mitochondrial mass^50^. c-Myc induces expression of glutaminase^38^, the first anaplerotic enzyme in glutamine metabolism, and transcriptionally regulates expression of glutamine importers^39^. In addition, FLT3 inhibition decreases glycolysis, increasing the reliance on glutamine metabolism to supply the tricarboxylic acid (TCA) cycle, which in turn supplies oxidative phosphorylation (OXPHOS)^51^. Accordingly, in this study, we found that combined gilteritinib and CUDC-907 treatment downregulates amino acid and fatty acid metabolism, as indicated by the significantly decreased amino acid pools, carnitines pools, and TCA cycle metabolites (Supplementary Figures S4–6). These data suggest that the combination treatment suppresses the production of critical metabolic intermediates and energy from the central metabolic pathways, which is supported by the significant decrease of ATP levels in the cells (Supplementary Figure S7). These data also suggest that the combination treatment may affect the biosynthesis of macromolecules such as protein for cell proliferation. It is important to note that the significant decrease of amino acid pools in the cells treated with combined CUDC-907 and gilteritinib was unlikely due to rapid catabolism of these amino acids which would result in increased levels of cellular urea. Instead, our metabolomics data shows significant decrease of urea in the combination-treated cells (Supplementary Figure S8). Specifically, CUDC-907 treatment significantly reduced glutamine and glutamate, which was further decreased when treated in combination with gilteritinib, suggesting that the cooperative reduction of glutamine may contribute to the synergistic antileukemic activity. Besides ATP, combined treatment with CUDC-907 and gilteritinib also resulted in significant decrease of other nucleotides indicating that the combination therapy impairs DNA and RNA synthesis and DNA repair. Taken together, our metabolomics data suggest that CUDC-907 and gilteritinib cooperate in downregulating cellular metabolism, leading to synergistic antileukemic activity.

We are encouraged by our in vivo findings. Given the known efficacy of gilteritinib against FLT3-ITD AML in both clinical and preclinical contexts, it is perhaps unsurprising to see evidence of substantial in vivo efficacy. Compared to the control arm (median survival 43 days), mice in the gilteritinib arm survived more than twice as long (median survival 104 days). In a prior CUDC-907 monotherapy experiment conducted in the same MV4-11 xenograft model, median survival was 44 days in the CUDC-907 group vs 33 days in the control group^14^. However, those mice received CUDC-907 at the same dose as was used here (100 mg/kg) for 8 consecutive days, then had 4 days ‘off,’ and received it again for a further 6 consecutive days, for a total of 14 treatment days compared to this study, in which mice were treated with CUDC-907 5 days on and 2 days off, for a total of 20 treatments. It is possible that more sustained treatment may result in improved efficacy, but this point will need to be further elucidated in subsequent investigations. Mice treated with both CUDC-907 and gilteritinib achieved a significant increase in survival (median survival >190 days). The dramatic increase in survival among the combination treatment group provides striking evidence of this combination therapy’s potential translational relevance.

In summary, we determined that the combination of gilteritinib plus CUDC-907 potently induces apoptosis in both FLT3-ITD AML cell lines and primary patient samples, and significantly prolonged survival of NSGS mice bearing systemic MV4-11 leukemia. These effects are exerted by multiple, complimentary mechanisms involving modulation of FLT3 protein levels, inactivation of the MAPK/ERK and JAK2/STAT5 pathways, reduction of Mcl-1 and c-Myc, induction of Bim, and downregulation of cellular metabolites, leading to synergistic antileukemic activity against FLT3-ITD AML. Although synergistic antileukemic interactions between CUDC-907 and gilteritinib could also be detected in certain FLT3-wt AML cells, potentially due to downregulation of wild-type FLT3 by CUDC-907 and inhibition of the receptor tyrosine kinase by gilteritinib, the detailed molecular mechanism remains to be determined. CUDC-907 is currently being investigated in Phase I and II clinical trials for treating various malignancies (www.clinicaltrials.gov). In a Phase I study, initial assessment of patients with relapsed or refractory diffuse large B-Cell lymphoma, including “double-expresser” lymphoma, showed very promising antitumor activity^52^. Importantly, CUDC-907 was well tolerated in elderly patients (median age 63, range from 22 to 83 years old)^52^. Furthermore, the safety and tolerability were further confirmed in the expanded phase I trial in DLBCL^53^. Our preclinical data along with the demonstrated safety and efficacy of gilteritinib in AML and CUDC-907 in DBCLC support further investigation of this promising combination therapy for the treatment of FLT3-ITD AML.

## Supplementary information

Supplemental Figures and Tables
