# Supplementary material for: The combination of CUDC-907 and gilteritinib shows promising in vitro and in vivo antileukemic activity against FLT3-ITD AML
Source: Blood Cancer J. 2021 Jun 7;11(6):111. doi: 10.1038/s41408-021-00502-7 (PMC8184771; doi:10.1038/s41408-021-00502-7)
Supplement: Supplementary file 1 — Supplemental Figures and Tables [file 41408_2021_502_MOESM1_ESM.pdf]

Figure S1

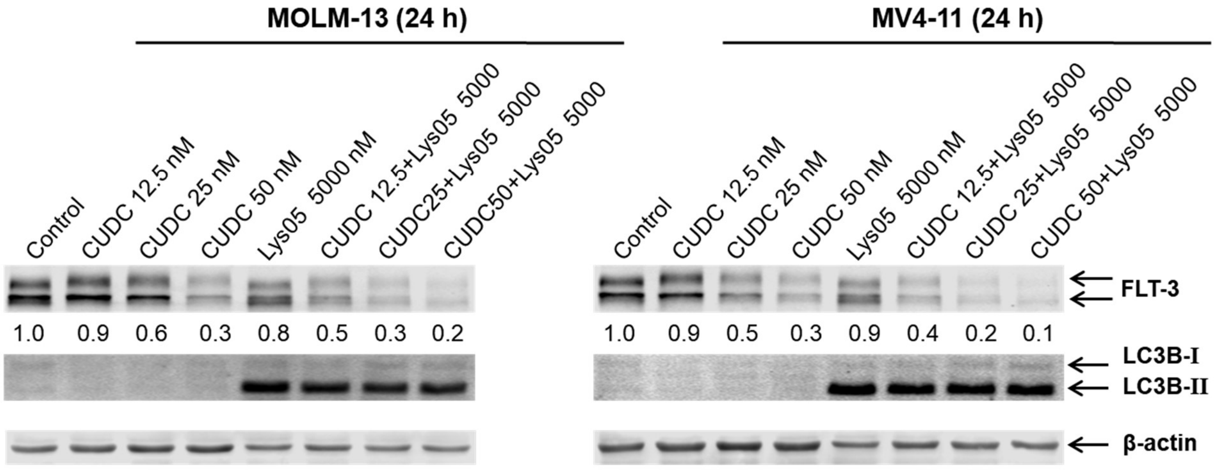

Figure S1. The lysosome inhibitor Lys05 fails to rescue FLT3 protein from CUDC-907 treatment in FLT3-ITD AML cell lines. MOLM-13 and MV4-11 cells were treated with vehicle control, varying concentrations of CUDC-907, or in combination with Lys05 for 24 h. Whole cell lysates were subjected to western blotting analysis. Densitometry was assessed via comparison to vehicle control and normalized to β-actin.

Figure S2

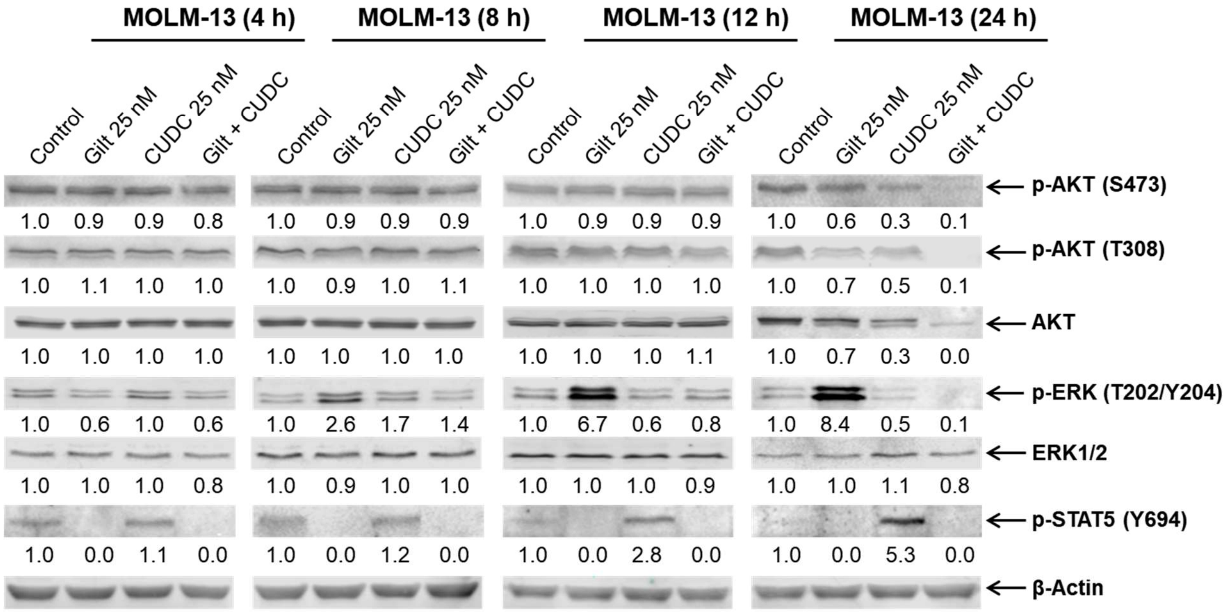

Figure S2. CUDC-907 and gilteritinib induce complimentary and cooperatively altered activity within the MAPK/ERK and JAK/STAT pathways. MOLM-13 cells were treated with gilteritinib, CUDC-907, both, or neither for 4, 8, 12, or 24 hours. Western blots were generated utilizing whole cell lysates, with representative blots shown, and densitometry displayed below each blot. Densitometry was assessed via comparison to vehicle control and normalized to β-actin.

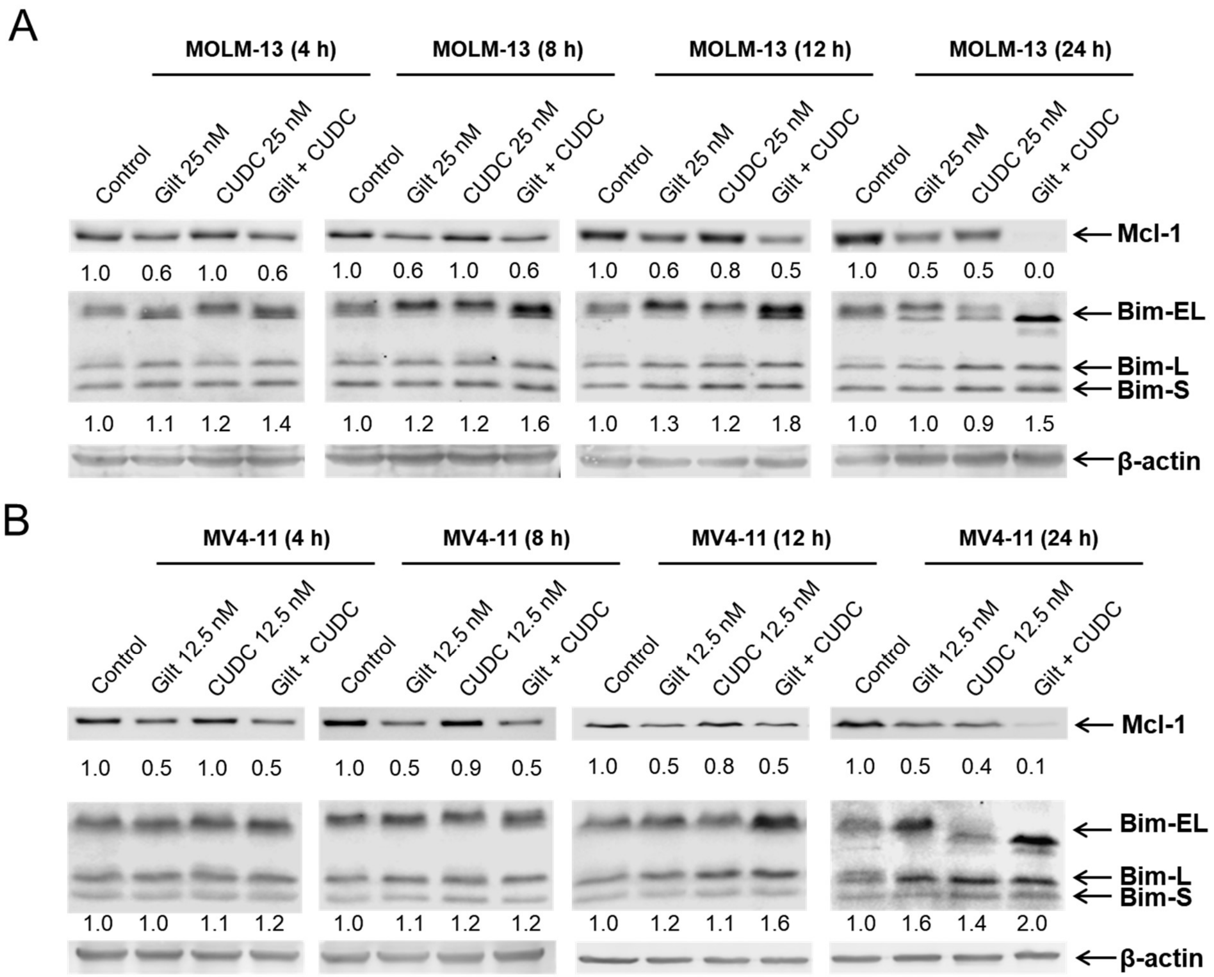

Figure S3. Time-dependent downregulation of Mcl-1 and concurrent upregulation of Bim by CUDC-907 and gilteritinib. MOLM-13 (panel A) and MV4-11 (panel B) cells were treated with gilteritinib, CUDC-907, both, or neither for 4, 8, 12, or 24 hours. Western blotting analysis was performed to assess Mcl-1 and Bim protein levels. Densitometry measurements, normalized to β-actin and relative to control, are shown below the corresponding blot.

Figures S4-S8. CUDC-907 and gilteritinib treatment cooperatively decreases TCA cycle metabolites, amino acids, urea, nucleotides, and carnitines. MV4-11 cells were treated with gilteritinib and/or CUDC-907 for 8 hours, at concentrations of 12.5 nM. Cells were collected, washed with PBS, and cell pellets were stored at -80°C. Metabolites were quantitatively profiled using LC-MS/MS based targeted metabolomics platform in the Karmanos Cancer Institute Pharmacology Core. Data analysis was performed using www.MetaboAnalyst.ca, version 4.0. Amino acids (Figure S4), carnitines (Figure S5), TCA cycle metabolites (Figure S6), nucleotides (Figure S7), and urea (Figure S8) were significantly downregulated in the combined gilteritinib and CUDC-907 treatment compared to vehicle control and single drug treatment. *indicates p<0.05 compared to control and #indicates p<0.05 compared to control, CUDC-907, and gilteritinib.

Figure S4

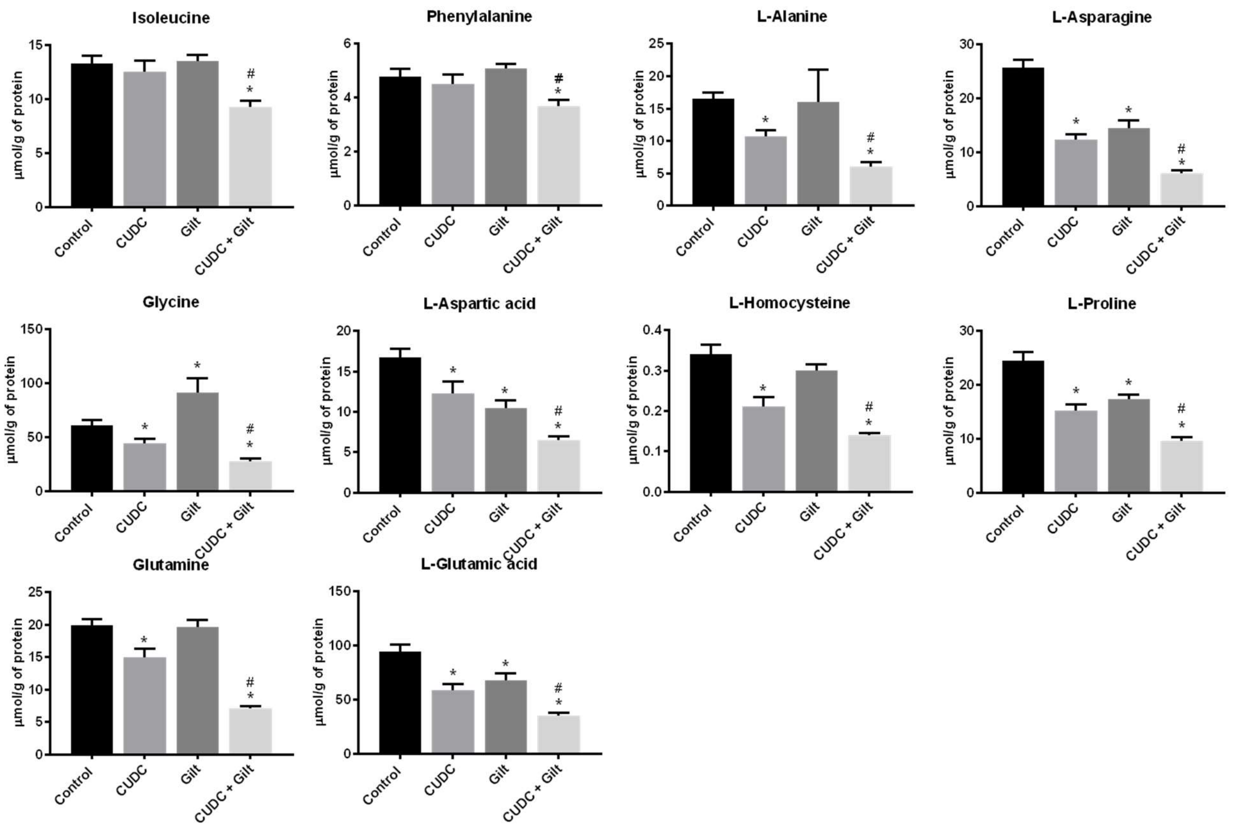

Figure S5

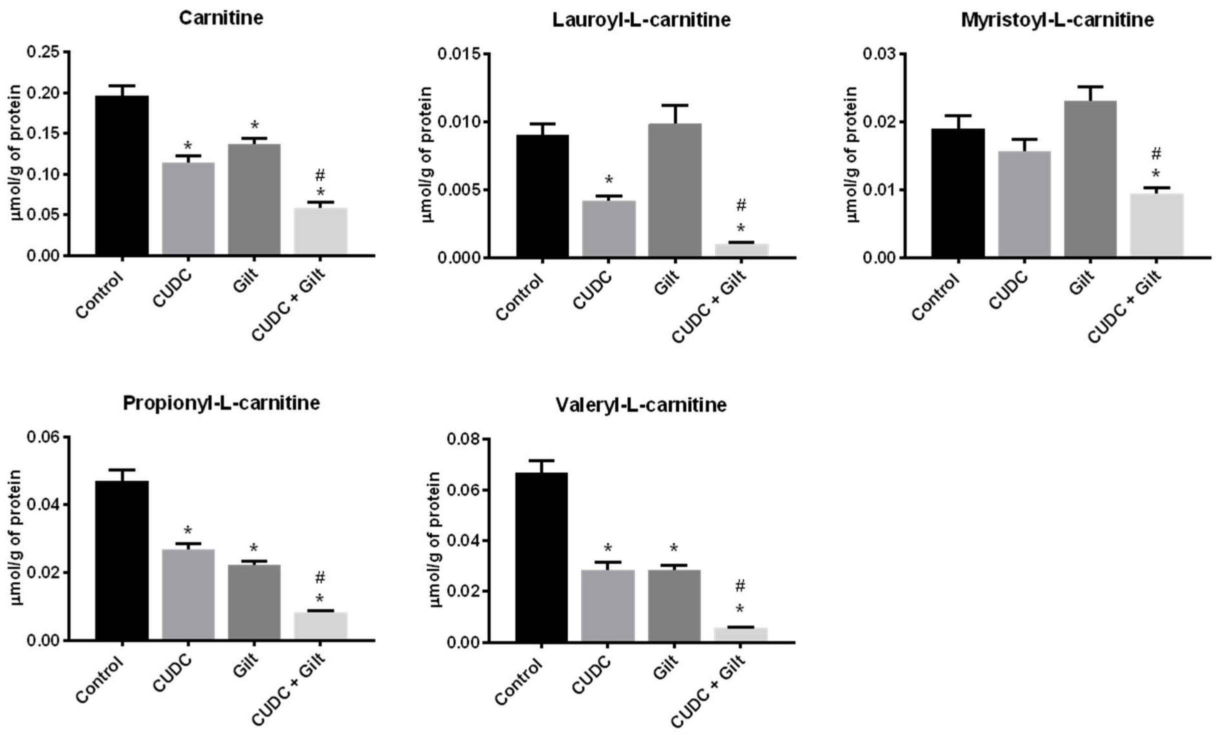

Figure S6

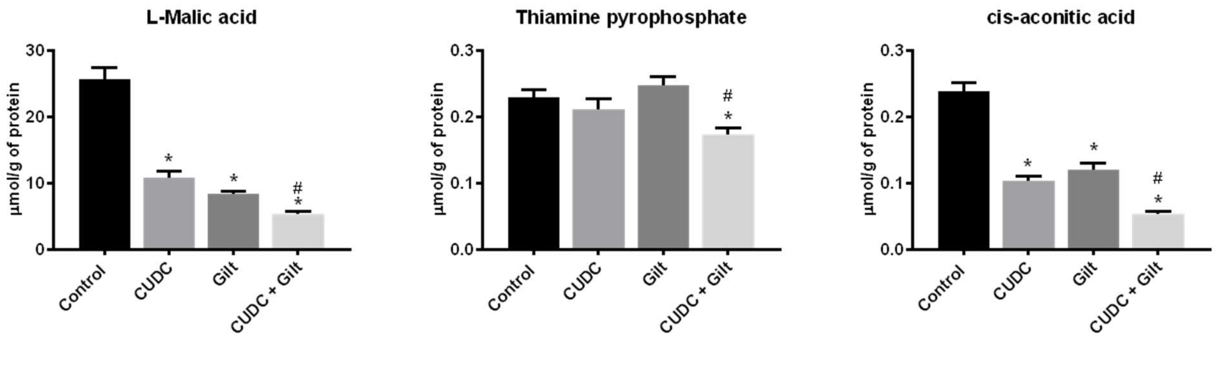

Figure S7

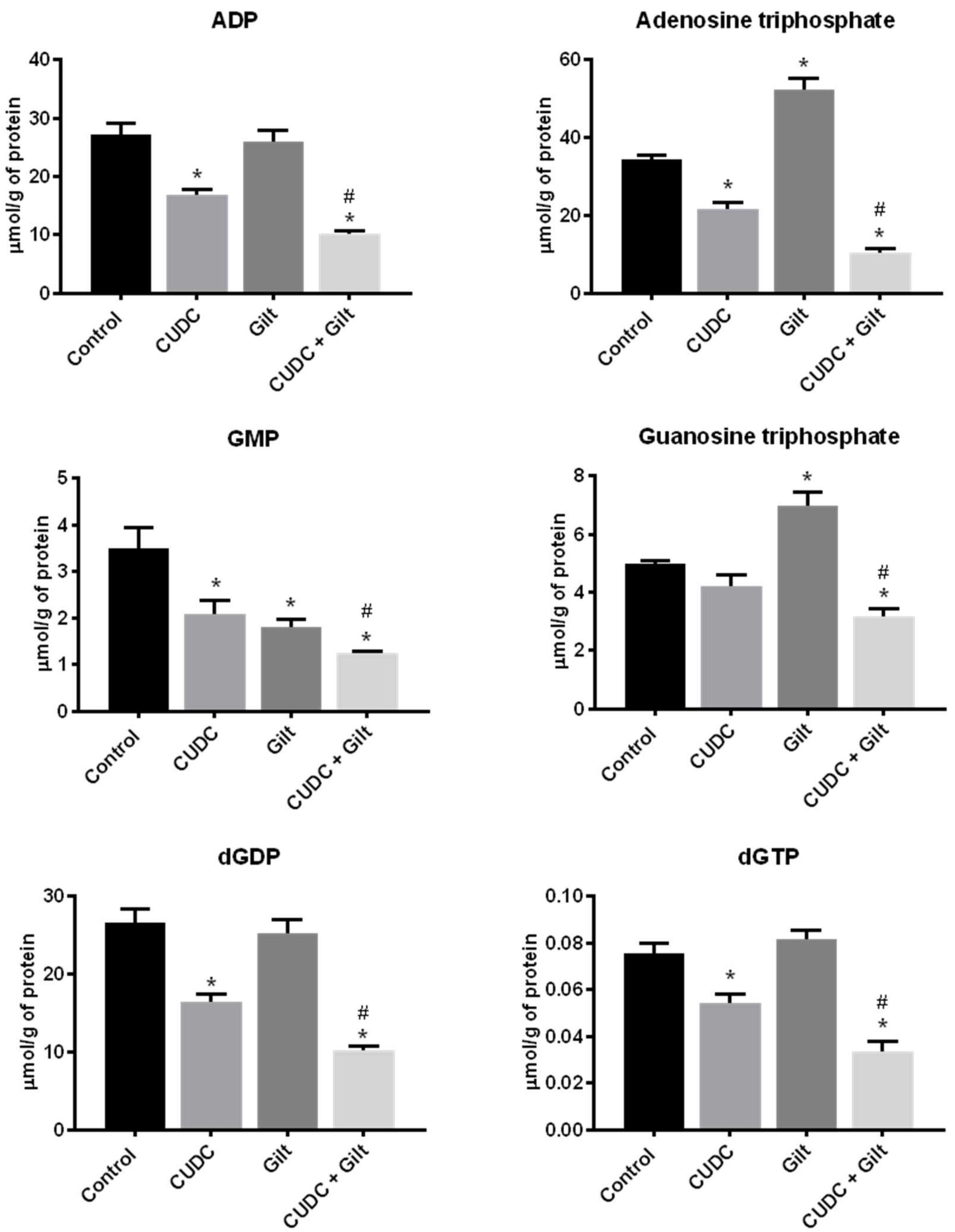
ADP
Adenosine triphosphate
GMP
Guanosine triphosphate
dGDP
dGTP
µmol/g of protein
Control
CUDC
Gilt
CUDC + Gilt

Figure S8

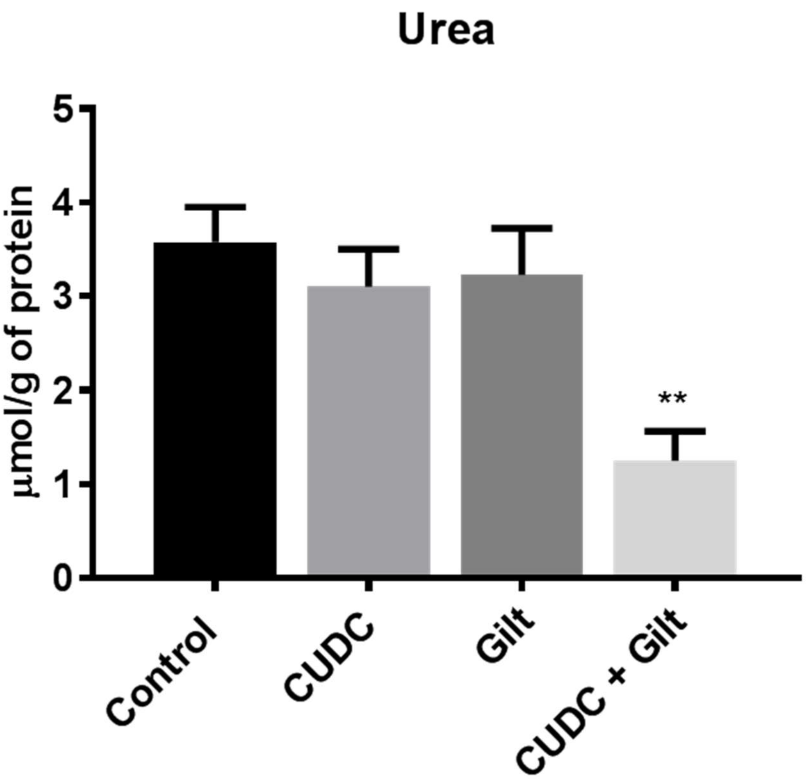

Table S1. Patient characteristics of primary AML patient samples

Patients	Gender	Age (year)	Disease status	FAB subtype	Cytogenetics	Blast purity (%)	Gene mutation
AML#170	Female	50	newly diagnosed	M4	46, XX, t(3; 3)(q21; q26)	72.0	SF3B1
AML#180	Male	68	Relapsed	M5	46, XY	96.5	FLT3-ITD, NPM1,DNMT3A
AML#207	Female	66	Newly diagnosed	M3	46, XX, t(15; 17)(q24; q21)	92.0	FLT3-ITD, PML-RARα
AML#208	Female	54	Newly diagnosed	M2	46, XX	88.0	FIT3-ITD, IDH2, NPM1, TP53, ZRSR2
AML#210	Female	1	Newly diagnosed	M4/5	46, XX, t(1; 11)(q21; q23)	80.5	
AML#212	Male	11	Newly diagnosed	M2	NA	63.0	FLT3-ITD
AML#213	Male	3	Newly diagnosed	M4/5	46, XY	64.0	FLT3-ITD
AML#214	Male	8	Newly diagnosed	M2	46, XY	86.5	FLT3-ITD
AML#216	Female	14	Newly diagnosed	M2	46, XX	96.5	GATA2, NPM1
AML#235	Female	33	Newly diagnosed	M4/5	46, XX	87.0	NPM1
AML#254	Female	15	Newly diagnosed	M5	46, XX	78.0	MLL-AF9, KMT2A, NF1
AML#256	Male	57	Newly diagnosed	M4/5	47, XY,+8	80.5	MLL-PTD, DNMT3A, SH2B3, FLT3-TKD, TCF, RUNX1
AML#257	Female	53	Newly diagnosed	M4/5	46, XX	94.5	FLT3-ITD, NPM1, TET2

Table S2. Differentially expressed metabolites (combination compared to control and single drug treatment)

Name	f.value	p.value	-log10(p)	FDR
Valeryl-L-carnitine	208.13	5.01E-13	12.3	8.0705E-11
Pantothenic acid	190	1.02E-12	11.992	8.2004E-11
Citicoline	144.03	8.68E-12	11.061	3.4951E-10
2-Ketohexanoic acid	134.47	1.47E-11	10.832	4.4615E-10
Propionyl-L-carnitine	132.35	1.66E-11	10.779	4.4615E-10
Uridine triphosphate	98.711	1.55E-10	9.8083	3.1289E-09
Lauroyl-L-carnitine	95.74	1.96E-10	9.708	3.5042E-09
Taurine	89.709	3.2E-10	9.4949	5.1514E-09
Cytidine triphosphate	86.422	4.24E-10	9.373	6.2001E-09
Adenosine triphosphate	84.604	4.97E-10	9.3037	6.284E-09
L-Malic acid	84.368	5.07E-10	9.2946	6.284E-09
Dihydroxyacetone phosphate	81.621	6.5E-10	9.187	7.4768E-09
cis-aconitic acid	72.176	1.63E-09	8.789	1.7447E-08
4-Hydroxyproline	67.716	2.61E-09	8.584	2.48E-08
UDP	66.855	2.86E-09	8.5429	2.5622E-08
uridine diphosphate glucose	63.602	4.14E-09	8.3834	3.3293E-08
Glutamine	62.92	4.48E-09	8.3491	3.4318E-08
Cystathionine	61.506	5.29E-09	8.2767	3.8701E-08
N-Acetyl-L-aspartic acid	60.455	6E-09	8.2219	4.1998E-08
L-Asparagine	58.572	7.56E-09	8.1215	5.0712E-08
Adenosine diphosphate ribose	57.578	8.56E-09	8.0673	5.5153E-08
Gluconic acid	55.405	1.13E-08	7.9458	7.0151E-08
Glutathione	53.208	1.52E-08	7.8185	9.0566E-08
ADP	49.387	2.6E-08	7.5853	1.4424E-07
dGDP	48.385	3.01E-08	7.5215	1.615E-07
Carnitine	46.584	3.95E-08	7.4037	1.9858E-07
CDP	42.438	7.65E-08	7.1165	3.621E-07
Folic acid	41.977	8.26E-08	7.0831	3.7993E-07
Thiamine	41.294	9.27E-08	7.0329	4.0338E-07
N-Methyl-D-aspartic acid	40.509	1.06E-07	6.9743	4.4948E-07
Aminoadipic acid	39.287	1.31E-07	6.8811	5.2925E-07
Pyridoxamine	37.059	1.97E-07	6.7045	7.5696E-07
NADP	35.417	2.7E-07	6.5684	9.6651E-07
Phosphorylcholine	34.317	3.36E-07	6.4741	1.1748E-06
Nicotinamide	33.781	3.74E-07	6.4272	1.2808E-06
N-Acetylglutamine	33.642	3.85E-07	6.415	1.2901E-06
Argininosuccinic acid	33.247	4.17E-07	6.3799	0.00000137
L-Homocysteine	32.185	5.2E-07	6.2838	1.6752E-06
L-Proline	31.643	5.84E-07	6.2336	1.8274E-06
Picolinic acid	31.55	5.96E-07	6.225	1.8274E-06
Creatine	29.224	9.98E-07	6.0009	2.9756E-06
dGTP	25.624	2.38E-06	5.6226	6.7345E-06
Glycine	24.826	2.93E-06	5.533	0.000008135
NAD	24.203	3.46E-06	5.4613	9.4326E-06
L-Glutamic acid	22.691	5.24E-06	5.281	0.00001405
Guanosine triphosphate	21.669	7.02E-06	5.1536	0.000018232
D-Sedoheptulose 7-phosphate	8.9794	0.001015	2.9938	0.0018352
N-Acetylglutamic acid	21.131	8.23E-06	5.0846	0.000021031
L-Aspartic acid	18.461	1.9E-05	4.7205	0.000047145
beta-nicotinamide D-ribonucleotide	17.784	2.39E-05	4.6219	0.000058265
GMP	17.082	3.04E-05	4.5165	0.000072076
Myristoyl-L-carnitine	16.593	3.62E-05	4.4411	0.000080987
Malonyl CoA	16.345	3.96E-05	4.4023	0.000087344
6-phosphogluconate	14.238	8.84E-05	4.0537	0.00018745
Sarcosine	13.745	0.000108	3.9668	0.00022027
Taurodeoxycholic acid	12.547	0.000179	3.7464	0.00036085
Methionine sulfoxide	12.03	0.000225	3.6469	0.00044273
L-Alanine	10.309	0.000509	3.2933	0.00097544
Isoleucine	9.4619	0.000784	3.1055	0.0014684
Asymmetric dimethylarginine	9.1018	0.00095	3.0224	0.0017374
Creatinine	8.4919	0.001327	2.8772	0.0023474
Deoxyribose 5-phosphate	7.3442	0.00259	2.5867	0.0042991
Urea	6.6404	0.004018	2.396	0.0065336
Thiamine pyrophosphate	6.3928	0.004716	2.3265	0.0075169
Phenylalanine	6.0442	0.00594	2.2262	0.0091953
N-Acetyl-L-alanine	5.5168	0.00853	2.0691	0.012485

Table S3. Pathway analysis of the differentially expressed metabolites (combination compared to control and single drug treatment)

Pathway Name	Match Status	p	-log(p)	Holm p	FDR	Impact
Arginine biosynthesis	6/14	6.179E-6	11.994	5.1903E-4	5.1903E-4	0.2335
Aminoacyl-tRNA biosynthesis	9/48	5.4517E-5	9.817	0.0045249	0.0016028	0.0
Alanine, aspartate and glutamate metabolism	7/28	5.7241E-5	9.7682	0.0046938	0.0016028	0.64263
Nicotinate and nicotinamide metabolism	5/15	1.6391E-4	8.7162	0.013277	0.0034422	0.46053
Purine metabolism	9/65	6.1796E-4	7.3891	0.049437	0.010382	0.11537
Glyoxylate and dicarboxylate metabolism	5/32	0.0066118	5.0189	0.52234	0.092566	0.12963
Pyrimidine metabolism	5/39	0.015354	4.1764	1.0	0.17398	0.09797
Nitrogen metabolism	2/6	0.019996	3.9122	1.0	0.17398	0.0
D-Glutamine and D-glutamate metabolism	2/6	0.019996	3.9122	1.0	0.17398	0.5
Glutathione metabolism	4/28	0.020711	3.8771	1.0	0.17398	0.36615
Thiamine metabolism	2/7	0.027303	3.6007	1.0	0.2085	0.66667
Glycine, serine and threonine metabolism	4/33	0.035851	3.3284	1.0	0.25096	0.3387
Citrate cycle (TCA cycle)	3/20	0.039332	3.2357	1.0	0.25414	0.1512

Table S4. Combination index values for Figures 2A, 2B, 2D, and 2G

		Combination Index			
Figure 2A	MOLM-13	Gilt 12.5 nM + CUDC 12.5 nM	Gilt 25 nM + CUDC 12.5 nM	Gilt 12.5 nM + CUDC 25 nM	Gilt 25 nM + CUDC 25 nM
		0.252	0.119	0.256	0.157
	MV4-11	Gilt 12.5 nM + CUDC 12.5 nM	Gilt 25 nM + CUDC 12.5 nM	Gilt 12.5 nM + CUDC 25 nM	Gilt 25 nM + CUDC 25 nM
		0.350	0.113	0.319	0.141
Figure 2B	MOLM-13	CUDC 12.5 nM + Gilt 12.5 nM	Gilt 25 nM + CUDC 12.5 nM	Gilt 12.5 nM + CUDC 25 nM	Gilt 25 nM + CUDC 25 nM
		0.212	0.116	0.113	0.096
	MV4-11	CUDC 12.5 nM + Gilt 12.5 nM	Gilt 25 nM + CUDC 12.5 nM	Gilt 12.5 nM + CUDC 25 nM	Gilt 25 nM + CUDC 25 nM
		0.240	0.346	0.330	0.519
Figure 2D	MV4-11/AraC-R	Gilt 12.5 nM + CUDC 12.5 nM	Gilt 25 nM + CUDC 12.5 nM	Gilt 12.5 nM + CUDC 25 nM	Gilt 25 nM + CUDC 25 nM
		0.102	0.082	0.153	0.150
Figure 2G	THP-1	Gilt 1000 nM + CUDC 50 nM	Gilt 1000 nM + CUDC 100 nM	Gilt 2000 nM + CUDC 50 nM	Gilt 2000 nM + CUDC 100 nM
		0.734	0.874	0.626	0.965
	OCI-AML3	Gilt 1000 nM + CUDC 25 nM	Gilt 1000 nM + CUDC 50 nM	Gilt 2000 nM + CUDC 25 nM	Gilt 2000 nM + CUDC 50 nM
		0.419	0.149	0.267	0.050

Table S5. Combination index values for Figure 3A

	Combination Index			
AML#180	Gilt 1000 nM + CUDC 12.5 nM	Gilt 2000 nM + CUDC 12.5 nM	Gilt 1000 nM + CUDC 25 nM	Gilt 2000 nM + CUDC 25 nM
	0.323	0.375	0.564	0.528
AML#207	Gilt 1000 nM + CUDC 25 nM	Gilt 1000 nM + CUDC 50 nM	Gilt 2000 nM + CUDC 25 nM	Gilt 2000 nM + CUDC 50 nM
	0.009	0.01	0.009	0.005
AML#208	Gilt 500 nM + CUDC 12.5 nM	Gilt 500 nM + CUDC 25 nM	Gilt 1000 nM + CUDC 12.5 nM	Gilt 1000 nM + CUDC 25 nM
	0.022	0.008	0.041	0.010
AML#212	Gilt 2000 nM + CUDC 25 nM	Gilt 2000 nM + CUDC 50 nM	Gilt 4000 nM + CUDC 25 nM	Gilt 4000 nM + CUDC 50 nM
	0.219	0.264	0.186	0.229
AML#213	Gilt 2000 nM + CUDC 25 nM	Gilt 2000 nM + CUDC 50 nM	Gilt 4000 nM + CUDC 25 nM	Gilt 4000 nM + CUDC 50 nM
	0.372	0.514	0.292	0.406
AML#214	Gilt 1000 nM + CUDC 25 nM	Gilt 1000 nM + CUDC 50 nM	Gilt 2000 nM + CUDC 25 nM	Gilt 2000 nM + CUDC 50 nM
	0.207	0.248	0.204	0.244
AML#257	Gilt 1000 nM + CUDC 50 nM	Gilt 1000 nM + CUDC 100 nM	Gilt 2000 nM + CUDC 50 nM	Gilt 2000 nM + CUDC 100 nM
	0.12	0.177	0.036	0.054

Table S6. Combination index values for Figure 3C

	Combination Index			
AML#170	Gilt 4000 nM + CUDC 50 nM	Gilt 4000 nM + CUDC 100 nM	Gilt 8000 nM + CUDC 50 nM	Gilt 8000 nM + CUDC 100 nM
	0.176	0.236	0.104	0.324
AML#210	Gilt 2000 nM + CUDC 25 nM	Gilt 2000 nM + CUDC 50 nM	Gilt 4000 nM + CUDC 25 nM	Gilt 4000 nM + CUDC 50 nM
	0.309	0.185	0.233	0.114
AML#216	Gilt 1000 nM + CUDC 25 nM	Gilt 1000 nM + CUDC 50 nM	Gilt 2000 nM + CUDC 25 nM	Gilt 2000 nM + CUDC 50 nM
	3.847	1.996	2.189	0.834
AML#235	Gilt 1000 nM + CUDC 50 nM	Gilt 1000 nM + CUDC 100 nM	Gilt 2000 nM + CUDC 50 nM	Gilt 2000 nM + CUDC 100 nM
	0.509	0.642	0.465	0.84
AML#254	Gilt 1000 nM + CUDC 50 nM	Gilt 1000 nM + CUDC 100 nM	Gilt 2000 nM + CUDC 50 nM	Gilt 2000 nM + CUDC 100 nM
	0.683	0.95	0.742	1.038
AML#256	Gilt 1000 nM + CUDC 50 nM	Gilt 1000 nM + CUDC 100 nM	Gilt 2000 nM + CUDC 50 nM	Gilt 2000 nM + CUDC 100 nM
	0.787	1.441	0.889	1.698
